# Structural Studies of HNA Substrate Specificity in Mutants of an Archaeal DNA Polymerase Obtained by Directed Evolution

**DOI:** 10.3390/biom10121647

**Published:** 2020-12-08

**Authors:** Camille Samson, Pierre Legrand, Mustafa Tekpinar, Jef Rozenski, Mikhail Abramov, Philipp Holliger, Vitor B. Pinheiro, Piet Herdewijn, Marc Delarue

**Affiliations:** 1 Unit of Structural Dynamics of Biological Macromolecules, UMR 3528 du CNRS, Institut Pasteur, 25–28 rue du Dr Roux, 75015 Paris, France; camille.samson160591@gmail.com (C.S.); tekpinar@buffalo.edu (M.T.); 2 Division of Biological Sciences, Synchrotron SOLEIL, 91190 Saint Aubin, France; pierre.legrand@synchrotron-soleil.fr; 3 KU Leuven, Rega Institute for Medical Research, Medicinal Chemistry Herestraat, 49-box 1041, 3000 Leuven, Belgium; jef.rozenski@kuleuven.be (J.R.); mikhail.abramov@kuleuven.be (M.A.); vitor.pinheiro@kuleuven.be (V.B.P.); piet.herdewijn@kuleuven.be (P.H.); 4 MRC Laboratory of Molecular Biology, Francis Crick Avenue, Cambridge CB2 0QH, UK; ph1@mrc-lmb.cam.ac.uk

**Keywords:** DNA polymerase, protein expression and purification, xeno-nucleic acid (XNA), crystallography, structural biology

## Abstract

Archaeal DNA polymerases from the B-family (polB) have found essential applications in biotechnology. In addition, some of their variants can accept a wide range of modified nucleotides or xenobiotic nucleotides, such as 1,5-anhydrohexitol nucleic acid (HNA), which has the unique ability to selectively cross-pair with DNA and RNA. This capacity is essential to allow the transmission of information between different chemistries of nucleic acid molecules. Variants of the archaeal polymerase from Thermococcus gorgonarius, TgoT, that can either generate HNA from DNA (TgoT_6G12) or DNA from HNA (TgoT_RT521) have been previously identified. To understand how DNA and HNA are recognized and selected by these two laboratory-evolved polymerases, we report six X-ray structures of these variants, as well as an in silico model of a ternary complex with HNA. Structural comparisons of the apo form of TgoT_6G12 together with its binary and ternary complexes with a DNA duplex highlight an ensemble of interactions and conformational changes required to promote DNA or HNA synthesis. MD simulations of the ternary complex suggest that the HNA-DNA hybrid duplex remains stable in the A-DNA helical form and help explain the presence of mutations in regions that would normally not be in contact with the DNA if it were not in the A-helical form. One complex with two incorporated HNA nucleotides is surprisingly found in a one nucleotide-backtracked form, which is new for a DNA polymerase. This information can be used for engineering a new generation of more efficient HNA polymerase variants.

## 1. Introduction

DNA polymerases (DNAPs) are essential for genome replication, and also for the maintenance of its integrity [1]. No life would be possible without DNAP activity. The role of DNAP is to catalyze the incorporation of nucleotides in the 5′ to 3′ direction to a growing DNA strand, called the primer strand, using a DNA template strand as a guide [2].

A common overall shape is observed in all DNAPs, which is reminiscent of a right hand that grips the DNA with both the fingers and the thumb, while the palm domain contains the catalytic site and the two magnesium ions [3]. Based on primary amino acid sequence similarities, DNAPs were classified into different families, which are named A, B, C, D, X, Y, and reverse transcriptase (RT) [4,5,6]. Nearly all DNAPs belong either to the Klenow-fold (family A, B, Y and RT), or to the Polβ-fold (family C and X) [7]. The D-family is an exception and contains a catalytic two-beta-barrel domain that is different from both the Klenow and Polβ folds but that is found in all multi-subunit RNA polymerase [8]. Usually, DNAP need at least two catalytic magnesium ions in their active site to be functional [9]. However, the presence of a third metal ion is required for the eukaryotic B-family DNAP δ [10] and the archaeal DNAP (KOD and 9°N) [11], as well as for the X-family Polβ and Polµ [12]. These catalytic ions are coordinated in an octahedral fashion by three strictly conserved aspartates that belong to well-identified sequence motifs, called A- and C-motifs [4,13].

DNAPs play a central role not only in DNA replication and repair but also in biotechnology [14]. For example, polymerase chain reaction (PCR) is one of the most important applications of DNAPs, making use of thermostable A- or B-family DNAPs, from bacteria and archaea respectively [15]. In addition, due to their capacities to accept modified nucleotides, DNAPs are now widely used in synthetic biology, especially archaeal polymerases from the B-family that have been shown to be able to accept modified DNA substrates, such as xenobiotic nucleic acids (XNA).

XNAs are synthetic polymers that deviate from RNA and DNA but that can still carry genetic information [16]. Apart from this role, XNAs can fold and interact in vitro and in vivo with ligands and be used as nuclease-resistant aptamers. Clearly, XNAs could be a powerful tool to transmit or store genetic information orthogonal to natural systems [17,18]. XNAs could be classified into two groups, based on their structure. The first group is composed of XNAs that are modified on nucleobases. Chemical modifications can be added to N7 in purines or C5 in pyrimidines of the nucleobases [19,20] or a larger nucleobase expansion containing one or two benzene units can be studied [21,22]. In addition, an artificially expanded genetic information system, abbreviated as AEGIS, was developed. AEGIS adds nucleotide building blocks to the four usual ones found in standard DNA by shuffling hydrogen-bonding units on the nucleobases. Thus, AEGIS retains the overall Watson–Crick nucleobase pairing geometry [23]. Unfortunately, these artificial nucleotides do not bind to natural DNA and lose the ability to communicate with extant molecular biology [24]. The second group of XNAs, which contains alternative sugar rings or modified backbone linkages, retains the key capacity of Watson–Crick base-pairing with natural nucleic acids. However, even minor chemical changes can lead to a wide variety of helical conformations, duplex stability, and altered base-pairing preferences [25]. The ability to cross-pair with DNA and/or RNA is clearly dependent on the sugar moiety [26]. In some cases, XNAs hybridize strongly and selectively with complementary DNA or RNA. Naturally, these cases have been studied a lot during the last 10 years (TNA, PNA, HNA…). One of these cases is the 1,5-anhydrohexitol nucleic acid (HNA), which will be the subject of this article (Supplementary Materials Figure S1A).

HNA derives from a six-membered hexitol sugar ring. It is composed of 2′,3′-dideoxy-1′,5′-anhydro-D-arabino-hexitol nucleosides with 4′-6′ phosphodiester inter-nucleotide linkages, with the base positioned at the 2′-position. Model building predicts that an HNA-RNA duplex will fold into an A-helical conformation, with ribose adopting a 3′-endo sugar pucker, similar to RNA [27,28]. To confirm the presumed A-form character of HNA, a solution structure of a HNA oligomer bound to a complementary RNA oligomer was determined by nuclear magnetic resonance (NMR) [29] and a crystal structure by X-ray crystallography [27] (Supplementary Materials Figure S1B). These structures confirm that the hybrid duplex formed normal Watson–Crick base pairs and prefers the A-type form of the double helix.

XNAs can be synthesized and replicated chemically [30]. Nevertheless, chemical polymerization remains relatively inefficient and expensive. Therefore, new options need to be explored for their replication, such as enzymatic synthesis, which is, however, hindered by the stringent substrate specificity of DNAPs. Thus, efforts to overcome this concern led to the development of engineered polymerases, which can generate XNA from DNA, as well as DNA from XNA, by directed evolution techniques.

In particular, the compartmentalized self-tagging (CST) strategy enabled the discovery of polymerases capable of processive HNA synthesis [31]. This approach was performed on libraries of mutants of a variant of the replicative B-family DNAP, which was isolated from the archaeal hyperthermophilic Thermococcus gorgonarius (Tgo). This variant will be hereafter referred to as TgoT and contains mutations in the uracil-stalling function (V93Q), the 3′-5′ exonuclease function (D141A, E143A), as well as a “Therminator” mutation (A485L). One polymerase mutant, TgoT_6G12, was found to display DNA-templated HNA polymerase activity in the presence of all four hNTPs. TgoT_6G12 polymerase corresponds to TgoT variant with 14 supplementary mutations (V589A, E609K, I610M, K659Q, E664Q, Q665P, R668K, D669Q, K671H, K674R, T676R, A681S, L704P, and E730G). In addition, another TgoT mutant capable of synthesizing complementary DNA from an HNA template was discovered. This mutant, named TgoT_RT521, is a reverse transcriptase that was engineered de novo [31]. Compared to TgoT, this mutant has only two additional mutations, I521L and E664K.

Numerous apo X-ray structures of DNAPs are accessible in the protein data bank (PDB). However, available data for the B-family DNAPs in binary or ternary complexes with DNA are rare. Only two crystal structures of archaeal DNAPs complexed with the primer and template strands together with the incoming dNTP are available. These structures correspond to those from Thermococcus kodakaraensis (KOD) and Thermococcus sp.9°N-7 (9°N) [11]. As B-family DNAPs capable of binding to XNA could display strong biotechnological potential, a better characterization of the structures of XNA-polymerases (XNAPs) is required. For this reason, three crystal structures of two XNA-polymerases were recently solved involving TNA and PNA [32,33]. However, no crystal structure of HNA- TgoT variants are available yet.

In this study, six X-ray structures of DNA and HNA polymerases are reported: (i) three apo- structures of TgoT, TgoT_6G12, and TgoT_RT521-; (ii) two DNA-bound structures of TgoT_6G12 in binary and ternary complexes; and (iii) one DNA-bound structure of TgoT_6G12, which has incorporated two HNA nucleotides. In addition, using partial X-ray data coupled to in silico studies, we propose a model of the ternary structure of TgoT_6G12 complexed with a hybrid duplex (HNA primer-DNA template) that is stable in molecular dynamics simulations. Structural comparison of all complexes allows to delineate a set of interactions and conformational changes required to promote HNA synthesis.

## 2. Materials and Methods

### 2.1. TgoT, TgoT_6G12, TgoT_RT521 Polymerases

#### 2.1.1. TgoT DNA Polymerase

Here, a variant of the replicative polymerase of *Thermococcus gorgonarius* (TgoT) was used. This variant is characterized by three mutations, in the uracil-stalling site (V93Q), in the 3′-5′ exonuclease catalytic site (D141A, E143A), and a “Therminator” mutation (A485L).

#### 2.1.2. TgoT_6G12 DNA Polymerase

In addition to the four mutations already described for the TgoT variant, TgoT_6G12 is mutated on the following positions: V589A, E609K, I610M, K659Q, E664Q, Q665P, R668K, D669Q, K671H, K674R, T676R, A681S, L704P, E730G.

#### 2.1.3. TgoT_RT521 Polymerase

In addition to the four mutations already described for the TgoT variant, this third variant of the polymerase of *Thermococcus gorgonarius* is mutated on the following positions: I521L and E664K.

### 2.2. Purification

The three TgoT variants were cloned into pET29 vector, then overexpressed in *Escherichia coli* BL21 (DE3) Star, which were cultured in Luria Broth (LB) Medium supplemented by kanamycin (30 mg.L^−1^). After induction at an optimal density (OD) of 0.6 with isopropyl β-D-1-thiogalactopyranoside (1 mM), bacteria were grown overnight at 20 °C. Cell were harvested by centrifugation for 20 min at 4000× *g* and lysed by sonication using lysis buffer (25 mM Tris-HCl pH 7.5, 100 mM NaCl, 0.1 mM EDTA, 1 mM DTT, 5 mg lysozyme, and a cocktail of protease inhibitors). The cell debris were pelleted by ultracentrifugation for 30 min at 20,000× *g* and the supernatant was heated at 70 °C during 20 min. After a second centrifugation at 20,000× *g* for 30 min, the supernatant was loaded onto 5 mL HisTrap FF crude. The protein was eluted by increasing concentrations of imidazole from 0 to 500 mM using the buffer B (25 mM Tris-HCl pH 7.5, 100 mM NaCl, 0.1 mM EDTA, 1 mM DTT, and 500 mM Imidazole). Before being loaded onto a Hitrap Heparin HP column, the elution fractions were pooled and imidazole concentration was decreased to 50 mM with buffer C (25 mM Tris-HCl pH 7.5, 200 mM NaCl, 0.1 mM EDTA, 1 mM DTT). The protein was eluted by increasing concentrations of NaCl with the high salt buffer B (25 mM Tris-HCl pH 7.5, 1 M NaCl, 0.1 mM EDTA, 1 mM DTT). Purest fractions were pooled and concentrated using 3 × 10^4^ kDa Vivaspin to a final volume of 1 mL. The purification was achieved by size exclusion chromatography (Superdex 200 HiLoad GE) pre-equilibrated with the buffer G (50 mM Tris-HCl pH 7.5, 200 mM NaCl, 0.1 mM EDTA, and 1 mM DTT). Purified protein was concentrated to 10 mg/mL^−1^ and frozen in liquid nitrogen. The samples were stored at −80 °C. Importantly, unlike TgoT and TgoT_6G12, the pH of each buffer used to purify TgoT_RT521 was increased from 7.5 to 8.5.

### 2.3. Protein Crystallography

Crystallization setups were done using the hanging drop vapor diffusion method. To obtain a final drop size of 2–2.5 µL, proteins were mixed with the reservoir solution in ratios 1:1, 1.5:1, and 1:1.5. Then, it was equilibrated against a 1-mL reservoir. Crystals were transferred into mother liquor containing 30% ethylene glycol concentrations in the respective crystallization conditions before freezing in liquid nitrogen.

#### 2.3.1. Duplex Preparation

In total, 0.2 mM of the primer and 0.2 mM of the template were incubated in annealing buffer (10 mM MgCl_2_, 50 mM Tris-HCl pH 8, 1 mM EDTA) for 10 min at 95 °C and stepwise cooled to room temperature overnight.

#### 2.3.2. TgoT DNA Polymerase

The TgoT variant has crystallized at a final concentration of 5 mg.mL^−1^ and after incubation with a 1.5× primer-template duplex P5-T13′ (P5: 5′ CGC AT 3′; T13′: 3′ GCG TAA ACG GCA A 5′), 2 mM MnCl_2_, and a 5 mM excess of ddTTP monomer. Crystals were obtained in 10% PEG 20 K and 0.1 M MES-NaOH pH 6.5. In these conditions, no DNA was observed in the crystals and the structure of the Apo-TgoT polymerase was solved.

#### 2.3.3. TgoT_RT521 DNA Polymerase

Apo-TgoT_RT521 was concentrated to 10 mg/mL^−1^ and crystallized in 25% PEG 400, 0.1 M sodium acetate pH4.6, and 0.2M MgCl_2_.

#### 2.3.4. TgoT_6G12 DNA Polymerase

Apo-TgoT_6G12 was concentrated to 10 mg.mL^−1^ and crystallized in 0.2 M calcium acetate, 0.1 M HEPES-HCl pH 7.5, 12% PEG 8K.

The binary complex was prepared by incubating the TgoT-6G12 variant, at a final concentration of 5 mg.mL^−1^ with a 1.5× primer-template duplex P5-T13 (P5: 5′ CGC AT 3′; T13: 3′ GCG TAA TCG GCA A 5′), for one hour at 4 °C. A 5 mM excess of ddTTP monomer was added to the final solution. Only the binary complex co-crystallized in these conditions. Crystals were grown in 10% PEG 20 K and 0.1 M MES-NaOH pH6.5.

The ternary complex was prepared by incubating the TgoT-6G12 variant, at a final concentration of 5 mg/mL^−1^ with a 1.5× primer-template duplex P5-T13′ (P5: 5′ CGC AT 3′; T13′: 3′ GCG TAA ACG GCA A 5′) and 2 mM MnCl_2_, for 30 min at room temperature. A 5 mM excess of ddTTP monomer was added to the binary complex and the solution was incubated at room temperature for 30 min. The complex co-crystallized in 5% PEG 20 K and 0.1M MES-NaOH pH 6.0.

The binary complex, in which two HNA nucleotides (hCTP) were trapped, was prepared by incubating the TgoT-6G12 variant, at a final concentration of 5 mg/mL^−1^ with a 1.5× primer-template duplex P9-T14 (P9: 5′ GGA GGG CAG 3′; T14: 3′ CCT CCC GTC GGT TA 5′) and 2 mM MnCl_2_, for 30 min at room temperature. A 5 mM excess of hCTP monomer was added to the binary complex and the solution was incubated at room temperature for 30 min. Finally, 5mM of ddCTP was added to the solution for 30 min at room temperature. This complex co-crystallized in 10% PEG 20 K and 0.1 M MES-NaOH pH 6.5.

Diffraction data were collected at the beamlines PROXIMA-1 and PROXIMA-2 at the synchrotron SOLEIL (Saint-Aubin, France). Diffraction data were processed with XDS [34]. The 3-D structures of the polymerase were solved by molecular replacement using Phaser in Phenix [35]. The coordinates of the Tgo WT polymerase (PDB: 1tgo) [36] were used as template. The resulting model was refined using phenix.refine and BUSTER [37] and alternate cycles of refinement and manual building performed with Coot [38].

### 2.4. Mass Spectrometry

#### 2.4.1. DNA Extraction

DNA was extracted directly from solution or from crystals. One volume of phenol:chloroform:isoamyl alcohol (25:24:1) was added to the sample, and vortex for approximately 20 s. After 5 min of centrifugation at 16,000× *g*, at room temperature, the upper aqueous phase was transfer to a fresh tube. Then, the sample was precipitated by adding 1 µL of glycogen, 20 µL of NH_4_OAc, and 150 μL of ethanol (100%). To precipitate all DNA from the sample, the tube was placed at −20 °C overnight. The day after, the sample was centrifugated for 30 min at 16,000× *g*, at 4 °C. After removing the supernatant, 150 μL of ethanol (70%) were added to the pellet and the sample was centrifugated at 4 °C, for 2 min, at 16,000× *g*. Finally, the cDNA pellet was resuspended in 300 µL of water.

#### 2.4.2. Mass Spectrometry

HPLC was performed on a capillary liquid chromatography system (Waters CapLC, Milford, MA, USA) with a C18 reversed-phase column (PepMap 0.5 × 15 mm, LC Packings, Amsterdam, The Netherlands) using a buffer containing *N,N*-dimethylaminobutane (DMAB, Acros, Geel, Belgium) and 1,1,1,3,3,3-hexafluoro-2-propanol (hexafluoro isopropanol, HFiP, Acros, Geel, Belgium). The solvent system consisted of acetonitrile 50% (vol/vol) (Fisher Scientific, Loughborough, UK) as the organic phase and DMAB 0.05% (vol/vol) with HFiP 1% (vol/vol) in water as the aqueous phase (pH 8.0). Oligonucleotides were eluted with a flow rate of 12 μL/min applying a gradient starting at 2% organic phase and increasing by 2% per minute for 15 min. The concentration of the oligonucleotide samples was ~50 μM and 0.5 μL of product was injected per run.

Electrospray ionization mass spectra were obtained in negative ion mode on a quadrupole/time-of-flight mass spectrometer (Waters Synapt G2 HDMS, Milford, MA, USA) equipped with a standard ionization source. The instrument resolution was 1.5 × 10^4^ (FWHM) and leucine enkephalin was used as lock mass calibrant. Masses for the oligonucleotides were obtained by deconvolution of the spectra using the MaxEnt algorithm of the software (Waters MassLynx 4.1, Milford, MA, USA).

Under these experimental conditions, the primer and template are separated and the mass for both can be determined. Here, 50% of the template showed an extra hCTP attached to it, which is a feature of a terminal nucleotidyl transferase activity. The primer strand appeared to have incorporated two hCTP nucleotides (100%), as expected given the first templating bases (GG).

### 2.5. Molecular Dynamics (MD) Protocol

#### 2.5.1. System Preparation

The X-ray structure of TgoT_6G12 ternary complex was used to initiate the MD simulation. Missing residues of two large loops 664–692 and 705–716 were modelled from the 5VU9 structure [32], which has 91% sequence identity to the initial structure. MultiSeq tool (The Luthey-Schulten Group–University of Illinois at Urbana-Champaign) [39] in VMD [40] was used for alignment of the two structures. Double-stranded DNA in the initial structure was replaced with HNA-DNA double strand using the HNA-RNA 2BJ6 structure as the initial model [27] and manual structural alignment in Coot. Ribonucleotides in the 2BJ6 structure were converted to deoxynucleotides by simply replacing the 2′OH atoms by 2′H. After these preparations, the system was solvated in a truncated octahedral box with 13 Å minimal distance to the edges. The TIP3P water model [41] was preferred for simulating the solvent. Na+ and Cl− ions were added to neutralize the box and the ion concentration was set to 0.150 M. The final system composition is given in Table 1.

The position of O6 atom of 5′ end of the template DNA strand was restrained during the simulation with a harmonic force constant of 1000 kJ mol^−1^ nm^−2^. This atom is one of the farthest DNA atoms from the active site. Moreover, the distance between the 3′ end of DNA and 5′ end of the HNA was restrained with a harmonic potential, so as to preserve distance between the last base-pair. This distance restraint was preferred over a positional restraint to give the DNA-HNA complex a chance to adapt to conformational changes occurring in the system while keeping the free ends of the nucleic acids intact.

#### 2.5.2. MD Simulations

All of the simulation preparations were performed with AmberTools version 2019 (Department of Chemistry, Lensfield Road, Cambridge). Amber ff14SB force field [42] was used for the protein and Parmbsc1 force field [43] was used for the DNA. Previous HNA parameters [44], which were parameterized in accordance with Amber force field, were used to simulate HNA molecules. Final Amber topology and coordinates were converted to Gromacs format with ParmEd tool (http://parmed.github.io/ParmEd). Periodic boundary conditions were applied in all directions and the Particle Mesh Ewald (PME) method [45,46] was used for all electrostatic calculations. The LINCS algorithm was used to constraint hydrogen bonds [47,48]. Since all hydrogen bonds were constrained, the time-step was set to 2 femtoseconds for all of the simulations. Gromacs-2018 MD simulation package [49,50] was employed for the simulations.

The system was minimized for 5 × 10^4^ steps with 100 kJ mol^−1^ nm^−1^ force tolerance. The system satisfied the minimization condition within approximately 1 × 10^4^ steps. A two-step equilibration protocol was applied after the minimization. In the first step, the system was equilibrated under NVT ensemble for 50 ps. This was followed by a 5-ns-long equilibration under constant pressure and temperature (NPT). In the NPT equilibration step, the pressure was kept constant at 1 bar with Berendsen barostat [50] while the temperature was kept constant at 300 K with the velocity rescaling algorithm [51]. Then, we performed a 0.5-microsecond-long production run under conditions identical to the NPT equilibration step.

#### 2.5.3. HNA-DNA Duplex Conformation Analysis

Ideal A-form DNA and B-form DNA structures were needed to study the conformations adopted by HNA-DNA duplex. For this purpose, NAHELIX program was used to build an ideal A-form DNA of 13 nucleotides with 2.56 Å rise per base pair along the axis and 32.7° rotation per base pair [52]. The same procedure was repeated for an ideal B-form DNA of 13 nucleotides with 3.38 Å rise per base pair along the axis and 34.3° rotation per base pair. These ideal structures were used as references for RMSD analyses of HNA-DNA duplex. Helical twist and helical rise per base pair were measured with 3DNA program [53,54].

## 3. Results

### 3.1. Structure Analysis of the Apo Form of Three Tgo Polymerase Variants

To better understand how the mutations added on TgoT variants impact the structure of the polymerase, the X-ray structures of the apo form of TgoT, replicative polymerase TgoT_6G12, and reverse transcriptase TgoT_RT521 were solved by crystallography. Crystals were obtained for the three polymerases and diffracted at 2.4, 3.1, and 2.35 Å, respectively (Table 2).

**Figure 1:**
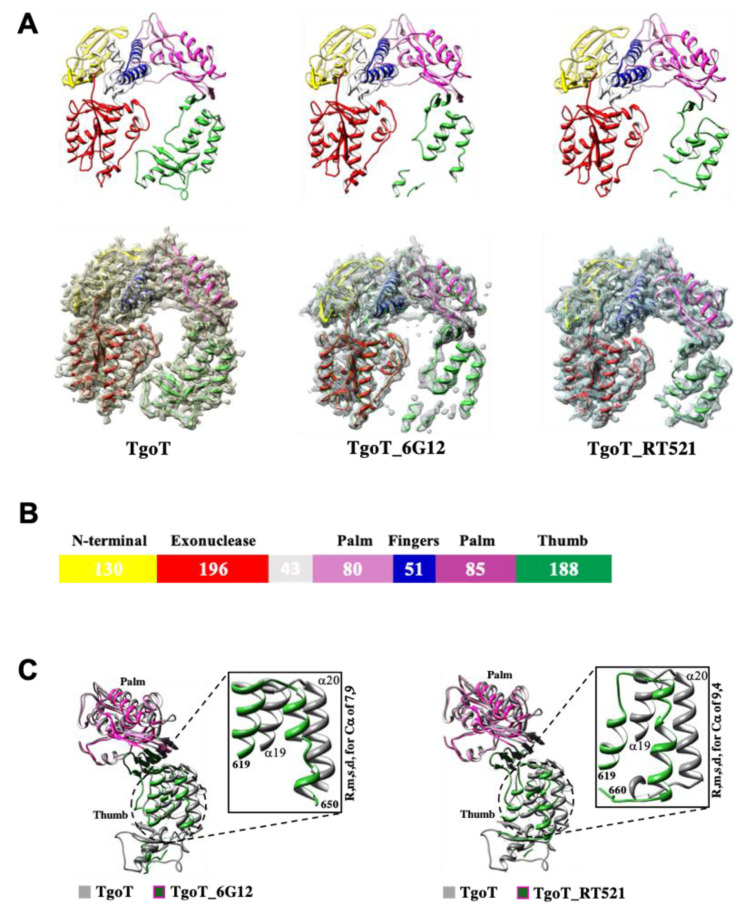
Structural comparison between TgoT apo form and its two variants, TgoT_6G12 and TgoT_RT521. (**A**) Cartoon representation of TgoT, TgoT_6G12, and TgoT_RT521 polymerases with a colored representation of domains (Upper panel). The domains are colored as follows: N-terminal domain (yellow), 3′-5′ exonuclease domain (red), palm domain (light and dark magenta for its N-terminal and C-terminal parts, respectively), fingers domain (blue), and thumb domain (green). An interhelical segment between the exonuclease and the palm domains is colored in light grey. The 2Fo–Fc X-ray electron density omit map is shown and contoured at a level of 1 σ (Down panel) as a grey mesh to illustrate the less ordered character of the thumb domain (green). (**B**) Domain organization in Tgo polB. Conserved domains are represented as colored boxes and their length is indicated. A non-conserved inter-helical segment is colored in light grey. (**C**) Overlay of the palm (light and dark magenta) and the thumb (green) domains of TgoT variants with the palm and thumb domains (grey) of TgoT. Broader movements observed in the thumb domains are highlighted with a zoom on helices S and T (r.m.s. deviation for Cα of 7.9 Å for TgoT_6G12 and 9.4 Å for TgoT_RT521).

Consistent with all other DNAPs from B-family, TgoT and its variants fold into five distinct structural subdomains (Figure 1A). Here, the N-terminal domain is represented in yellow, the exonuclease domain in red, and the catalytic domain composed of the palm, the fingers, and the thumb subdomains are represented in magenta, blue, and green, respectively. Comparison of TgoT_6G12 with TgoT_RT521 structures shows a strong similarity between the two polymerases (r.m.s. deviation for Cα atoms: 0.6 Å). Only few movements were observed in the thumb subdomains. The final apo TgoT_6G12 and TgoT_RT521 structures contained flexible segments in the thumb subdomains (Figure 1A). The high flexibility of these segments probably results from the absence of DNA or HNA inside the active site.

Next, the structures of TgoT_6G12 and TgoT_RT521 were compared to the one of TgoT. Slight movements in the finger domain (r.m.s. deviation for Cα atoms: 1.18 Å for TgoT_RT521 and 1.1 Å for TgoT_6G12) and in the exonuclease domain (r.m.s. deviation for Cα atoms: 0.8 Å for TgoT_RT521 and 1.0 Å for TgoT_6G12) were observed for both mutants. On the contrary, larger rigid-body movements in the palm domains (r.m.s. deviation for Cα atoms: 2.7 Å for TgoT_RT521 and 2.3 Å for TgoT_6G12) were observed (Figure 1C). As TgoT_6G12 and TgoT_RT521 structures contained unbuilt regions in the thumb subdomains, no value associated to the entire thumb region from TgoT versus those from each mutant was calculated. However, very large rigid-body movements can be locally seen between the thumb subdomain of TgoT and those of TgoT_6G12 and TgoT_RT521 (Figure 1C). For example, between amino acids 619 to 660, for TgoT_RT521, the r.m.s. deviation for Cα is 9.4 Å and between amino acids 619 and 650 for TgoT_6G12, it is 7.9 Å (Figure 1C). The helices α19 and α20 are part of these regions. Hence, overall, the crystal structure of the wild-type TgoT appears more stable than the two variants studied here.

### 3.2. TgoT_6G12 in Binary and Ternary Complexes with DNA

TgoT_6G12 is known to be able to bind both DNA-DNA natural duplex and HNA-DNA hybrid duplex. However, structural data on the TgoT_6G12 in binary or ternary complexes with DNA are missing. These data would be informative to better understand how to design additional mutations that could further enhance HNA binding and polymerase activity.

#### 3.2.1. Crystallization

To provide structural insights into the mechanism of DNA synthesis by an HNA-polymerase, the structure of TgoT_6G12 bound to a DNA primer-template (P-T) duplex was solved by X-ray crystallography. Co-crystals of the complex ready to accept an incoming dNTP were obtained and diffracted to 2.8 Å (Table 2). In a second experiment, crystals including an incoming nucleotide trapped inside the polymerase active site were generated. The crystals of the ternary complex of TgoT_6G12 diffracted to 3.2 Å, with two molecules per asymmetric unit (Table 2).

#### 3.2.2. Conformational Changes

**Figure 2:**
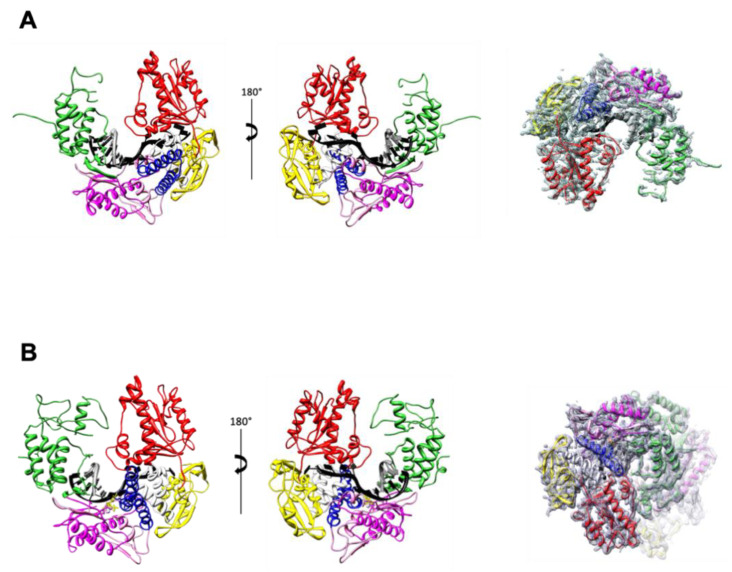
TgoT_6G12 polymerase structure in binary and ternary complexes. Overall structure of TgoT_6G12 in binary (**A**) and ternary complex (**B**). On the left, front and back views of the polymerase are colored by domain. The primer and template DNA strands are colored in grey and black, respectively. The domains are labeled as follows: N-terminal domain (yellow), 3’-5’ exonuclease domain (red), palm domain (light and dark magenta represent the N-terminal and the C-terminal respectively), fingers domain (blue) and thumb domain (green). An interhelical segment between the exonuclease and the palm domain is colored in light grey. On the right, the 2Fo–Fc electron density omit map is shown and contoured at a level of 1 σ to illustrate which domains are less ordered than the others. A rotation of 180° around a horizontal axis was applied between the middle panel and the right-hand panel.

Consistent with other solved structures of DNAP from B-family, the structures of the binary (Figure 2A) and ternary complexes (Figure 2B) show that the primer-template DNA duplex is bound in a groove formed by the palm and the thumb subdomains.

Archaeal polymerases from KOD and Tgo are two polymerases with 93.01% sequence identity (Supplementary Materials Figure S2). Since the structures of KOD polymerase in binary and ternary complex have been solved already [11,55], they were used here to help the analysis of TgoT_6G12 polymerase conformation. As observed for KOD polymerase, the thumb subdomain of TgoT_6G12 polymerase transitions from an ensemble of poorly defined conformations to a more well-ordered structure following P-T binding (Figure 2A,B). Still, in the binary complex structure, several parts of the thumb subdomain of TgoT_6G12 stayed poorly defined inside the electron density map (662 to 700, 706 to 716, and 763 to the C-terminal end (Figure 2A)). However, the thumb region in the ternary complex is mainly ordered. Indeed, only one internal region from amino acid 666 to 690 as well as the C-terminal part from amino acid 763 to the end are poorly defined (Figure 2B).

**Figure 3:**
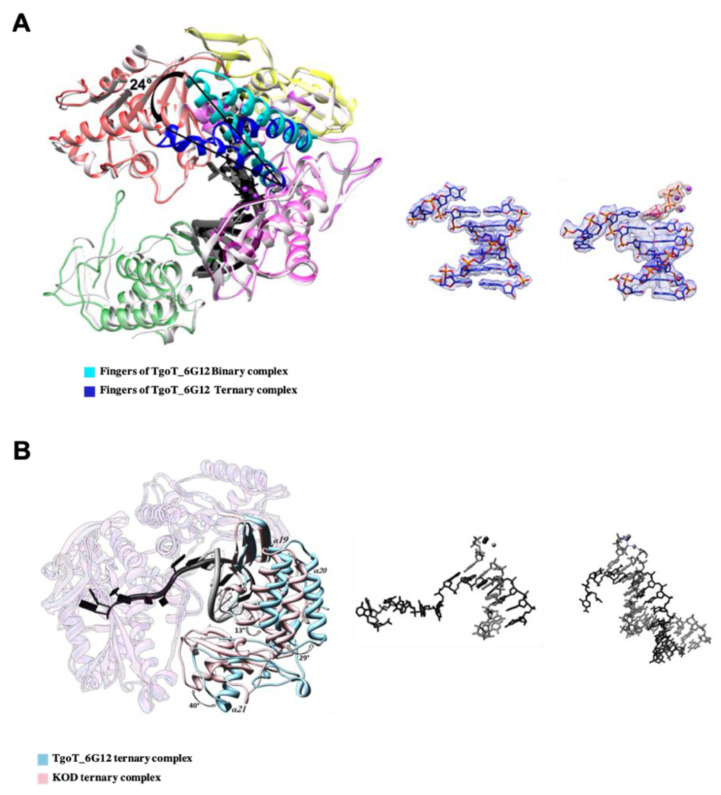
TgoT_6G12 polymerase conformational changes upon DNA and dNTP binding. (**A**) On the left, structural changes observed during nucleotide triphosphate binding are represented. The finger domain of the ternary complex (dark blue) is closed by approximatively 24° compared to the finger domain of the binary complex (cyan). On the right, the primer-template DNA duplex is represented, as observed in the binary and in the ternary complexes, respectively. The omit map electron densities of the primer-template, of the ddTTP, and of the three divalent ions are shown at 1 σ as a mesh and are labeled in blue for the primer-template and in pink for the ddTTP and the ions. (**B**) Comparison of the ternary structure of TgoT_6G12 and KOD polymerases. On the left panel, TgoT_6G12 polymerase is colored in light blue and KOD polymerase in light pink. The primer and template DNA strands are colored in grey and black, respectively. Helices α19, α20, and α21 of the thumb domain in TgoT_6G12 are tilted outward by 13°, 29°, and 40°, respectively. On the right panel, the DNA/DNA duplex is represented, as seen in TgoT_6G12 ternary complex (on the left) and in KOD ternary complex (on the right), as stick. The primer strands, the incoming nucleotides and ions are labeled in dark grey while the template strands are labeled in dark.

Another conformational change is observed with the ternary complex (Figure 3A). Contrary to the structure of TgoT_6G12 in the apo or binary complex, the finger domain of TgoT_6G12 in the ternary complex shows a closed conformation, indicated by a rotation of 24° of its two α-helices (α14 and α15). Similar observations were previously observed in the structure of KOD polymerase ternary complex [11]. In addition, the last resolved nucleotide of the template strand is stabilized by the N-terminal domain, as previously reported [11].

Superposition of the ternary structures of TgoT_6G12 and KOD reveals clear rigid body movements that are mainly located in the thumb subdomain (Figure 3B). In detail, helices α19 and α20 (a.a 618–632; a.a 639–652) of the TgoT_6G12 thumb subdomain tilted by 13° and 29° outward, respectively. In addition, a broader movement is observed for helix α21 (a.a 676–687), which is tilted by 40°. Lastly, helix α22 (a.a. 731–747) is tilted by 12°. The comparison between the two polymerases shows that the thumb subdomain of TgoT_6G12 polymerase displays a slightly less closed conformation than the one of KOD.

#### 3.2.3. DNA Interactions

**Figure 4:**
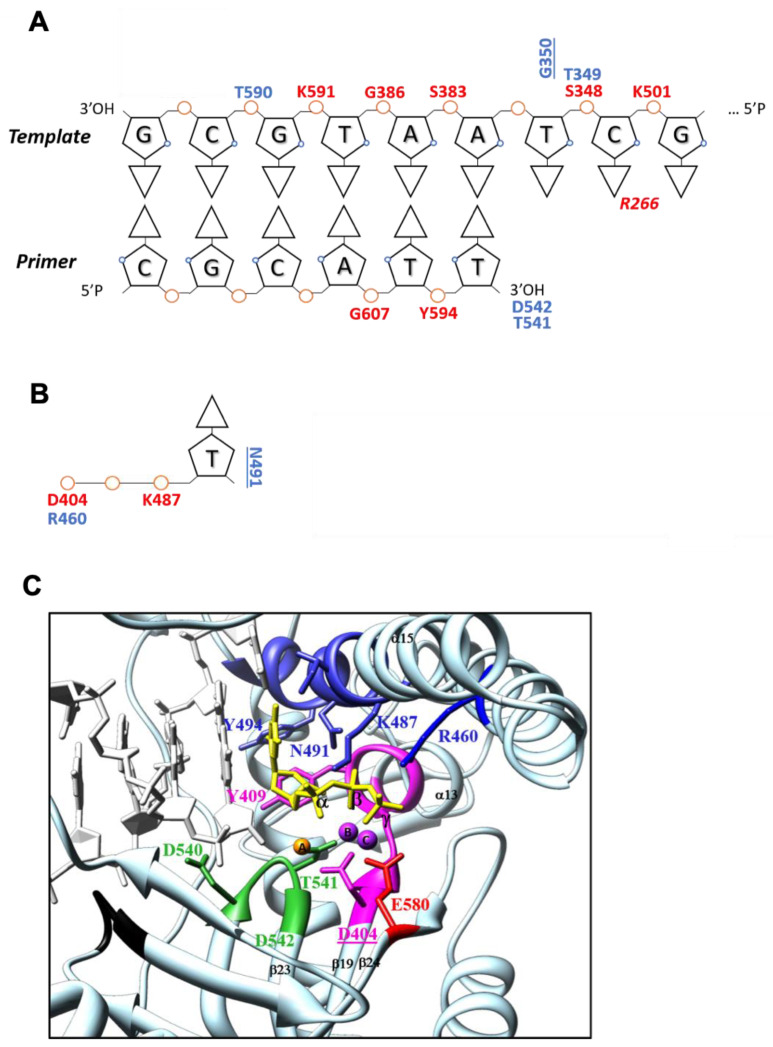
TgoT_6G12 active site analysis. (**A**) Pattern of interactions between TgoT_6G12 polymerase and the template/primer DNA duplex, in the binary complex, according to the strength of the interactions. The color code for the strength of interactions is: red for strong/moderate and direct interactions and blue for weak electrostatic but direct interactions. Interactions between the enzyme and the phosphate backbone of DNA are labeled above, for the template, and below, for the primer, each phosphate (represented by an orange circle). Interactions between the enzyme and the ribose moieties are labeled, underlined and vertical. Interactions between the polymerase and the nucleotide base are in italics. (**B**) Interactions of TgoT_6G12 with the ddTTP incoming nucleotide using the same labeling scheme. (**C**) TgoT_6G12 active site with the incoming ddTTP (colored in yellow). Representative sequence motifs are labeled. The Kx3NSxYGx2G B-motif in the finger domain is colored in dark blue. The A-motif (DxxSLYPSI) is colored in magenta and the catalytic residue D404 is underlined. The C-motif (DTDG) of the palm domain is colored in green and the KKKY motif of the thumb domain is colored in black. One other residue of the palm domain, which interacts with the ddTTP, is colored in red. The ddTTP makes contacts with residues of the palm and finger subdomains (D404, K487, R460, and N491). The Mg^2+^ in position A is colored in orange. It is coordinated by residues D404 (A-Motif) and D542 (C-Motif). The two Mn^2+^ are colored in purple. The Mn^2+^ in position B is directly coordinated by residues D404 and D542 and by the three α,β,γ phosphates of the ddTTP. The Mn^2+^ in position C is coordinated by residues E580 and D404 and by the γ-phosphate of the ddTTP.

TgoT_6G12-DNA interaction maps were created for the binary structure (Figure 4A) and the ternary structure (Supplementary Materials Figure S3). Most of the interaction involves the DNA phosphate backbone. For the binary structure, the hydrogen bonds involved in direct strong or moderate hydrogen bonds (2.2–3.2 Å) and direct weak electrostatic interactions (3.2–4 Å) were listed (Figure 4A). In addition, interactions between the incoming nucleotide and the TgoT_6G12 polymerase were listed (Figure 4B). As the chain F of the ternary structure appears to be far better ordered than the chain E, interactions between the incoming nucleotide and TgoT_6G12 polymerase were drawn from chain F. Some residues responsible for interaction with the DNA duplex are conserved throughout the entire B-family DNAPs. Especially, one residue that corresponds to the conserved motif KKKY (amino acids 591 to 594) of the thumb subdomain was observed to interact strongly with the phosphate backbone (residues K591). In addition, T541 and D542 residues, from the C-motif DTDG (amino acid 540 to 543), common to the whole archaeal B-family DNAP, were found to bind the primer 3′OH end (Figure 4A). All these interactions are consistent with the binary and ternary structure solved for KOD [11,55]. Only residues from amino acids 666 to 675, which make direct hydrogen bonds with the primer in KOD structures, are poorly defined inside the electron density map in the binary and ternary structure of TgoT_6G12.

#### 3.2.4. Active Site Analysis

The active site of TgoT_6G12 is composed of the helix α13 of the palm domain, the helix α15 of the finger domain, and the central β-sheet from strands 18 to 24 (Figure 4C), which contains the three highly conserved aspartate residues D404, D540, and D542 of the A- and C-motifs. These residues were shown to be the signature of the polymerase active site [56]. In the ternary structure of TgoT_6G12, three metal ions are observed at the active site, in the same position as observed with the structure of KOD in ternary complex (Mg^2+^ in position A, Mn^2+^ in position B and C, Figure 4C). The presence of the two manganese ions was determined by the anomalous signal. This observation confirms that the presence of three metal ions could be a characteristic for archaeal DNAPs. These ions are coordinated both by the enzyme and the incoming dNTP. Contrary to the ternary complex of KOD polymerase, the residue F405 is not interacting with metal ions (Figure 4C).

The incoming nucleotide (ddTTP) binding site contains two tyrosines: Y494 and Y409. In addition, Y409 forms the bottom of the binding site. ddTTP packs with its sugar moiety against Y409, as previously observed with the dATP in the structure of KOD polymerase ternary complex (Figure 4B,C). This explains the discrimination against ribonucleotides and constitutes the steric gate already described by other groups [11,57]. In addition, the pyrimidine base moiety of ddTTP makes contacts with N491, and several positively charged residues stabilize the triphosphate (Figure 4B).

### 3.3. TgoT_6G12 Binding Mode to the HNA-DNA Duplex: In Silico Approach

In the absence of crystals grown with an HNA-DNA heteroduplex, molecular dynamics simulations were conducted on a model of the ternary complex between TgoT_6G12 and an HNA-DNA hybrid duplex to understand the impact and importance of each TgoT_6G12 mutation. These simulations were based on our structure of TgoT_6G12 ternary complex. The HNA-DNA duplex was generated based on both the HNA-RNA (PDB: 2BJ6) structure solved by X-ray crystallography [27] and an unpublished HNA-DNA structure that we obtained using X-ray crystallography in which this duplex adopts an artefactual binding mode to TgoT_RT521; both conformations are highly similar. The post-reaction state is modeled with a bound HNA nucleotide (hNTP). An HNA primer of 11 nucleotides and a DNA template of 13 nucleotides were used.

A plot of Cα RMSD with respect to the initial frame in the production run is shown in the left panel of Supplementary Materials Figure S4A, indicating that an equilibrium was attained after about 20 ns. The RMSD saturates between 2 and 3 Å after this time. We also measured RMSDs of the HNA-DNA duplex with respect to the ideal A-form and B-form DNAs (right panel of Supplementary Materials Figure S4A). The duplex has a lower RMSD to the A-form compared to the B-form, which suggests that HNA-DNA duplex is more similar to the ideal A-form DNA duplex. Another issue worthy of investigation is the stability and interactions of two Mn2+ ions. These ions preserve an approximately 5 Å distance to each other throughout the simulation (left panel of Supplementary Materials Figure S4B). The first Mn2+ (Metal A) interacts strongly with E580 and D404. Moreover, it also forms strong interactions with E578 upon changes in the side chain orientation of E578 (middle panel of Supplementary Materials Figure S4B). The second Mn2+ (Metal B) maintains a stable distance of 2 Å with D404, which is a highly conserved residue, and it interacts with both oxygens of D542 (right panel of Supplementary Materials Figure S4B). Finally, we studied the stability of the nucleotide interactions: hNTP nucleotide preserves two hydrogen bonds with the DNA Adenine across the simulation (left panel of Supplementary Materials Figure S4C).

#### 3.3.1. HNA-DNA Hybrid Duplex

**Figure 5:**
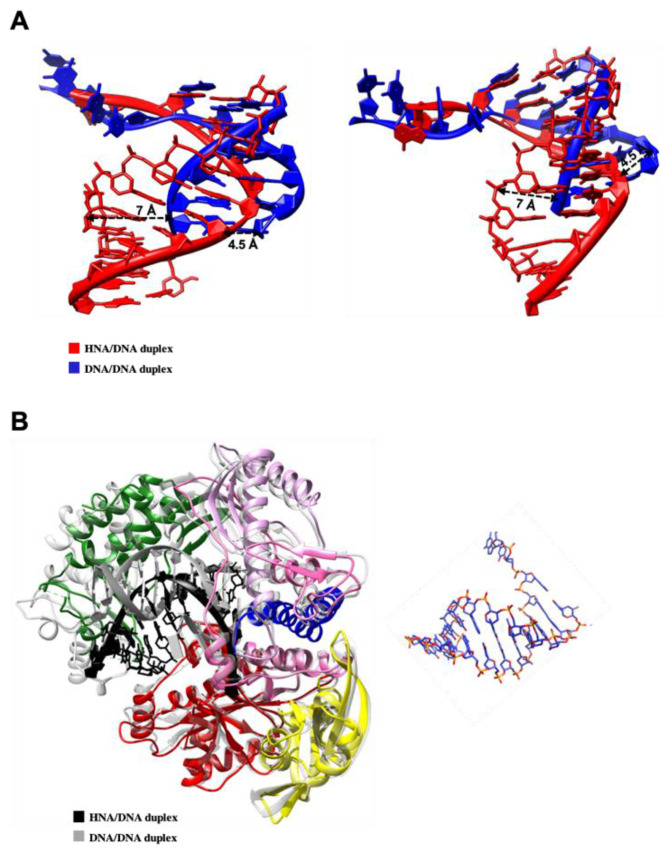
Comparison between a natural DNA-DNA duplex and a HNA-DNA duplex bound to Tgo_6G12. (**A**) Overlay of the HNA-DNA primer-template duplex (in red) with the natural DNA-DNA duplex (in blue), in two different orientations (left and right panels). Deviation between the two duplexes is labeled with dash lines. (**B**) On the left, superimposition of our X-ray ternary structure solved with a DNA/DNA duplex (colored by domains) with the in silico ternary structure modeled and equilibrated in the presence of an HNA/DNA duplex (in light grey). The DNA/DNA duplex is labeled in grey whereas the HNA/DNA duplex is labeled in black. On the right is represented the HNA/DNA duplex as stick, at the same scale.

In both structures of HNA-RNA duplexes solved by NMR and X-ray crystallography [11,33], the HNA-RNA duplex was found to form an A-type helical geometry. In our laboratory, a structure of an HNA-DNA duplex was also solved and confirms the A-form for a DNA-HNA duplex (data not shown). In the modeled structure of TgoT_6G12 bound to an HNA-DNA duplex, the A-form shape is retained. The HNA-DNA duplex stays closer to the A-form during the entire simulation (Supplementary Materials Figure S4A). In addition, all ribose groups adopt a 3′-endo sugar conformation in the HNA primer strand. During the simulation, the HNA-DNA hybrid duplex moves between 0 and 7 Å compared to the DNA-DNA duplex position (Figure 5A,B), due to differences in their helical geometry. Despite this large movement, the extremity of the HNA 3′OH primer remains located at the same position, as well as the DNA 3′ extremity, inside the active site.

#### 3.3.2. HNA-Specific TgoT Mutation Analysis in a Structural Context

Compared to TgoT, TgoT_6G12 variant is mutated on 14 residues (V589A, E609K, I610M, K659Q, E664Q, Q665P, R668K, D669Q, K671H, K674R, T676R, A681S, L704P, and E730G) to confer an HNA synthesis activity on the natural polymerase scaffold. In the X-ray ternary complex obtained in the presence of a DNA-DNA duplex, seven of the mutated residues were well defined, which corresponded to V589A, K659Q, E664Q, Q665P, A681S, L704P, and E730G. In addition, the main-chain atoms of two other residues were well located: E609K and I610M. However, five mutated residues (R668Q, D669Q, K674R, T676R, and K671H) were not observed in the final electron density map. To understand the impact of each mutation, observations done with the X-ray ternary structure of TgoT_6G12 were combined with the in silico model, in the presence of an HNA-DNA duplex. In addition, direct strong/moderate hydrogen bonds and weak electrostatic interactions between the phosphate backbone of the HNA-DNA duplex and TgoT_6G12 polymerase were listed (Supplementary Materials Figure S5). Concerning the active site, the same residues and motifs as those observed for TgoT_6G12 bound to a DNA-DNA duplex were observed (Supplementary Materials Figure S6).

**Figure 6:**
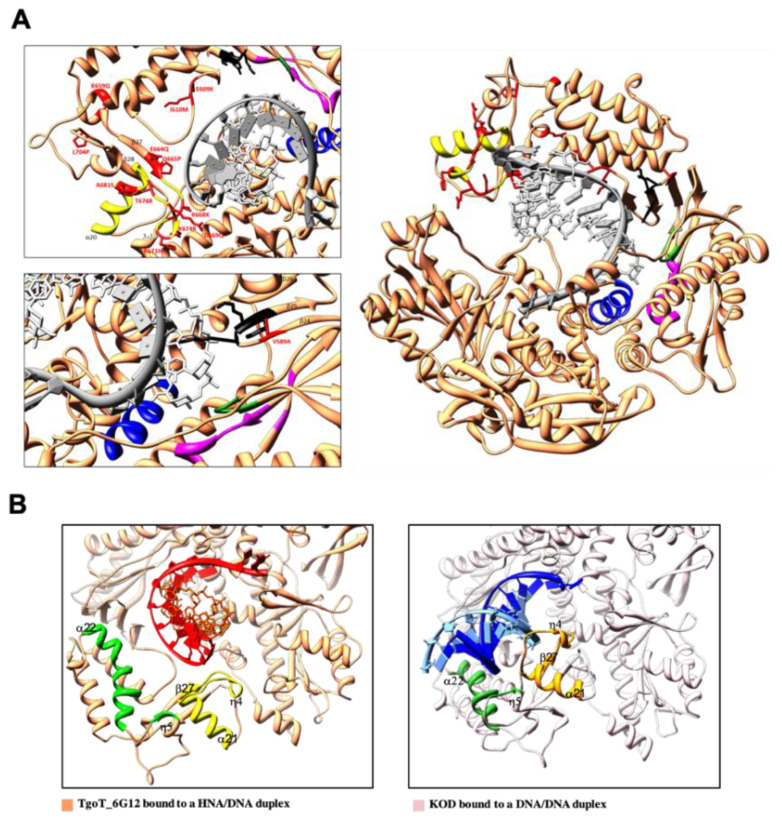
Analysis of TgoT_6G12 binding mode to the HNA-DNA duplex using MD simulations. (**A**) In the right panel, an overview of the closed complex of TgoT_6G12 polymerase (in coral) with an HNA-DNA heteroduplex. The template DNA strand is represented as a dark gray ribbon with its associated sugar/base backbone shown as a tube. The primer HNA strand is represented in light gray with atoms and bonds. On the left panel, a blow up of two regions of the right panel, but in a different orientation. Mutated residues that confer HNA binding and synthesis capacity are shown and labeled in red. The region from residues 664 to 688, which interacts with the template strand is labeled in yellow. Representative motifs are labeled. The A-motif (DxxSLYPSI), in addition to E578 and E580, is colored in magenta. The C-motif (DTDG) of the palm domain and the Kx3NSxYGx2G B-motif in the finger domain are colored in green and dark blue, respectively. The KKKY motif of the thumb domain, is labeled in dark. (**B**) Conformational changes observed between the ternary structure of TgoT_6G12 bound to a HNA-DNA duplex, on the left, and the ternary structure of KOD bound to a DNA-DNA duplex, on the right. The template strands of TgoT_6G12 and KOD are labeled in red and dark blue, respectively. The primer strands of TgoT_6G12 and KOD are labeled in orange and light blue, respectively. The region from amino acids 664 to 688 interacts with the template strand in TgoT_6G12 (yellow) and with the primer strand in KOD (orange). Helix η5 and α helix 22 are labeled in light and dark green, respectively, in TgoT_6G12 and in KOD ternary complexes. While these two helices are stabilizing the template strand in the KOD ternary structure, they are shifted away from the HNA-DNA duplex in TgoT_6G12 ternary structure.

All mutations are located inside the thumb subdomain and outside the active site pocket. Regarding the HNA primer strand, few residues make direct hydrogen contacts (Supplementary Materials Figure S5). In the in silico model of TgoT_6G12, residues T667, R668, V673, A675, R674, which are not visible in the X-ray structure, are now shown to interact with the DNA template strand (Figure 6A). In contrast, those residues are interacting with the DNA primer strand in the ternary structure of KOD, where the DNA is in a B-helical form.

In the structure, the strand β27 and the helix η4 (amino acid 664 to 676) surround and stabilize the 3′ end of the template strand (Figure 6A). Altogether, this region, highlighted in yellow in Figure 6A, contains eight of the mutated residues of TgoT_6G12. Strikingly, residue Q664, located on strand β27, has already been hypothesized to have a role in the specificity of the polymerase for the nascent duplex [58]. In addition, helix α21, which contains the mutated residue A681S, is also in proximity to the template strand (almost 7 Å).

Another residue, L704, located at the end of the strand β28, is mutated into a proline in TgoT_6G12 and could help the adaptation of the polymerase to the HNA substrate. Interestingly, in KOD ternary complex, the helix η5 that follows the strand β28 stabilizes the 5′ template strand (Figure 6B). Strong hydrogen bonds are observed between the phosphate backbone of DNA and residues R709, I710, and G711. However, in both ternary structures obtained for TgoT_6G12, this helix shifts by ~10 Å to avoid clashes with the DNA. We postulate that the proline 704 could be important to allow the movement of this helix. In addition, the 5′ template strand is stabilized by helix α22 in the ternary structure of KOD through residues Y731 and N735 that interact with the phosphate backbone of the DNA. In both ternary structures obtained for TgoT_6G12, helix α22 is tilted by 12° compared to its position in KOD ternary structure and is not stabilizing the template anymore (Figure 6B). Interestingly, helix α22 contains the mutated residue G730 that is present in TgoT_6G12.

The residue A589 (strand β24) may also be important in the DNA binding process because of its position in close proximity to the conserved sequence motif KKKY (residues 591 to 594). Indeed, the KKKY motif has already been shown to be essential for the stabilization of the B-form helix by keeping the primer and template close together [5]. In the in silico model, the HNA-DNA hybrid duplex is closer to an A-type double helix instead of a B-type double helix usually observed in DNA-DNA duplexes. As a consequence, the position of the nucleotide of the template that interacts with the KKKY motif shifts by almost 4 Å compared to its position in TgoT_6G12 bound to a DNA-DNA duplex (Figure 6A). Interestingly, strands β24, β25, and β26 (from amino acid 580 to 607), which contain the motif KKKY, shift by 4 Å toward the DNA template strand in the in silico model. We postulate that the mutation of the residue 589 could facilitate the interactions between the motif KKKY of the thumb domain and the nascent duplex.

To complement the observations done using the in silico approach, the missing parts of the X-ray ternary structure of TgoT_6G12 bound to a DNA-DNA duplex were also modeled (664–692 and 705–716) from 5VU9 structure and examined in the DNA-DNA context (Supplementary Figure S7). A rotation by almost 45° of the region containing strand β27, helix η4, and helix α21 was observed after comparison with the ternary structure of KOD (Supplementary Figure S7). As observed by the in silico approach, residues of this region interact with the template strand in the presence of an HNA-DNA duplex (Supplementary Materials Figure S8) but are located far from the DNA-DNA duplex in the crystal structure (Supplementary Materials Figure S8). In addition, this same region binds the primer strand in the ternary structure of KOD, not the template strand (Supplementary Materials Figure S8). Interestingly, a steric clash is observed if we superimpose the ternary structure of KOD and the modeled HNA-DNA duplex (Supplementary Materials Figure S8). Altogether, the region containing helix η5 and helix α22 of TgoT_6G12 rotate by almost 10° compared to its position in KOD ternary structure.

Together, crystal structures complemented by an in silico approach allows the proposal of a model of the ternary complex of TgoT_6G12 bound to a HNA-DNA duplex in the A-form, which helps to rationalize the majority of mutated residues, as they are indeed seen to be involved in the stabilization of the nascent hybrid HNA-DNA duplex in the A-form by interacting with the template strand. In particular, the mutations allow the establishment of new molecular interactions that are essential to position the nascent heteroduplex, as the local helical parameters of the DNA differ from those observed for a standard DNA-DNA P-T duplex.

### 3.4. One Nucleotide-Backtracked State for TgoT_6G12 after Two Successive HNA Synthesis

**Figure 7:**
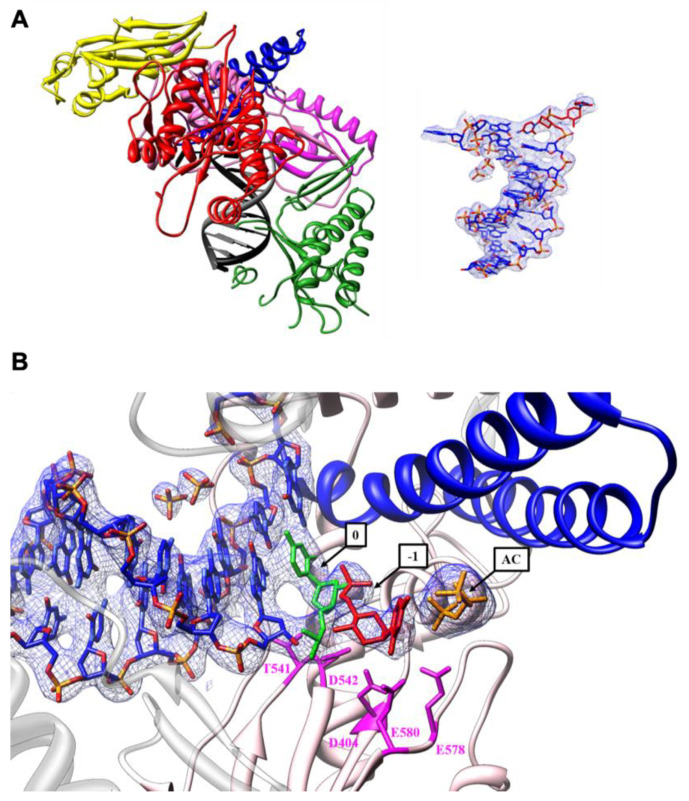
TgoT_6G12 trapped in a 1 nt-backtracked state after HNA synthesis. (**A**) Overall structure of TgoT_6G12 in binary complex, which has two incorporated hCs in the 3′end of the DNA primer. The primer and template strands are colored in grey and black, respectively. The domains are colored as indicated in Figure 1B. On the right, the electron density of the primer-template duplex is shown at 1 σ as a blue mesh. The two incorporated hCs are labeled in red. (**B**) TgoT_6G12 active site with two incorporated hCs, colored in green and red. The finger and palm subdomains are colored in blue and light pink, respectively. The two hCs make contacts with residues of the palm domain (D404, T541, D542, E578 and E580), labeled in magenta. The position of each hCTP is labeled by an arrow (Position 1 and 0). The additional triphosphate in the antechamber is colored in orange, labeled AC.

Finally, to provide structural insight into the mechanism of HNA synthesis, protein crystals were obtained under conditions designed to capture the first step in the HNA synthesis pathway. Before crystallization, TgoT_6G12 was incubated with a 9-mer primer (5′-GGAGGGCAG-3′), annealed to a 14-mer template (5′-ATTGGCTGCCCTCC-3′). In addition, DNA-protein complex was incubated with 5 mM of anhydrohexitol cytidine triphosphate (hCTP) first and subsequently with 5 mM of ddCTP, in order to block the polymerization reaction. Crystals were obtained and diffracted to 3.0 Å, with one molecule per asymmetric unit (Table 2). The X-ray structure reveals TgoT_6G12 in a binary complex, in pre-translocation state, with clear electron density for two additional nucleotides, one in the active site and one in an “entry” site similar to what is seen in a backtracked form already known for RNA polymerases (Figure 7). No electron density was observed for magnesium or manganese ions.

Mass spectrometry analysis was performed directly on protein crystals to characterize the polymerization product and confirm the analysis of the electron density. In a first step, DNA was extracted from dissolved crystals and all salts present in the DNA extract were removed. Then, the primer and the template strands were analyzed by mass spectrometry. The results clearly showed that two hCTPs were incorporated into the primer. In parallel, to confirm the results obtained with dissolved crystals, the same protocol was applied to the reaction products obtained in solution. DNA was extracted from a sample containing the polymerase, which was incubated with the same concentration of double-strand DNA as in the crystals, supplemented with hCTP and ddCTP. Both analyses gave similar results.

**Figure 8:**
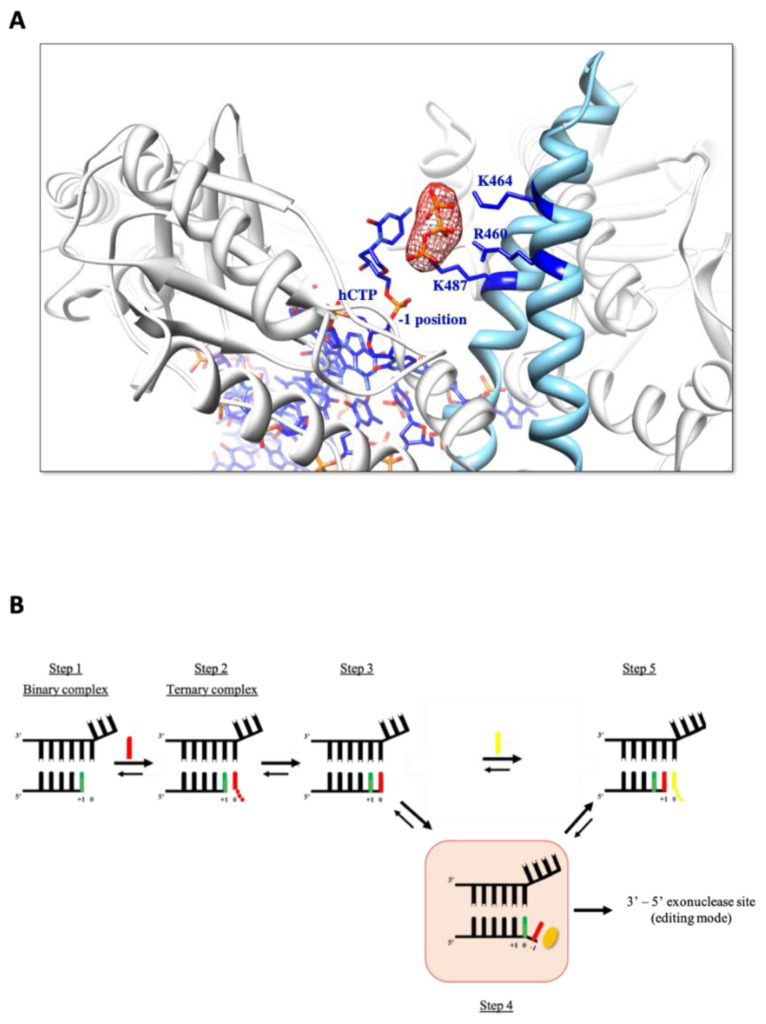
A possible 1 nt backtracking step in the catalytic cycle of Tgo_6G12. (**A**) X-ray structure of TgoT_6G12 in complex with a DNA substrate, which has incorporated two hCs at the 3′end of the primer. The 2Fo–Fc -electron density map (in red) is shown around an additional triphosphate and contoured at a level of 1 σ It forms a new dNTP site inside the polymerase, called the antechamber. The triphosphate makes contacts with positively charged residues of the finger subdomain (R460, K464 and K487), represented as sticks and labeled in blue. The hC in position −1 is labeled in blue. (**B**) Schematic view of the HNA synthesis pathway. An additional step in the catalytic cycle, step 4, is framed in red. The first incorporated hC is labeled in green and the second hC is labeled in red. The antechamber in represented by a yellow ellipse in step 4. Translocation leads to step 5 where a new incoming dNTP is represented in yellow, in position 0. Step 4 could serve as an intermediate step prior to send the fraying 3′ end to the exonuclease site, which is inactivated in TgoT mutants studied here.

In the X-ray structure, the open-position of the finger is compatible with a polymerase caught in a post-chemistry state complex, with a primer strand elongated by two hCs (Figure 7A). The first incorporated hC is located inside the active side and forms a C:G base pair with the template (Figure 7A,B). This hC is localized at the same position as the one of the incoming nucleotides observed in the ternary complex (position 0, labeled in green in Figure 7B). Two main residues make contacts with it: the two catalytic aspartate residues D540 and D542 (Figure 7B). The second hC was observed in an entry site (position −1, labeled in red in Figure 7B) distinct from the active site (position 0), without facing any templating base (Figure 7A, on the right). Several residues of the palm subdomain make contact with it. Hydrogen bonds are observed with two residues (E578 and E580) that normally stabilize the incoming ddTTP in the structure of the TgoT_6G12 in ternary complex. In addition, an additional triphosphate (labeled in orange on the Figure 7B) was observed in the electron density (Figure 8A), before the position of the second hCTP, in a site that we call the “antechamber” (position AC).

In conclusion, mass spectrometry analysis and X-ray structure show the existence of an unexpected binary complex upon addition of two successive hCs, which suggests a possible supplementary step in the classical kinetic model of this DNAP, called the backtracking mode. Moreover, an additional triphosphate group was observed, closed to the second hC nucleotide, in a new site (AC). The existence of a new dNTP binding pocket inside the polymerase as well as the unexpected binary complex that we obtained will be discussed in the next section with a tentative kinetic model.

## 4. Discussion

DNAPs are the primary enzymes necessary to faithfully replicate genetic material [59]. In particular, thermophilic archaeal DNAPs from the B-family have been shown to display high fidelity, processivity, and thermostability regarding DNA processing. Due to these characteristics, archaeal DNAPs from B-family have been widely used in many biotechnology applications [1]. Indeed, these polymerases have been exploited in many basic molecular biology reactions in DNA engineering processes, such as cloning, PCR, site-directed mutagenesis, or sequencing. In addition, B-family DNAPs from archaeal organisms have been shown to accept chemically modified nucleotides, such as xenobiotic nucleic acids [14,60]. For this purpose, engineered DNAPs, selected to accept XNAs, have been developed.

To illustrate how DNA and HNA are both recognized and selected by TgoT_6G12 and TgoT_RT521, a series of X-ray crystal structures and one in silico model are here provided. Binary and ternary structures from different hyperthermophilic DNAPs, such as the polymerase from Thermococcus kodakarensis, have been already determined by other groups and allow interesting comparisons [11,55]. However, only the WT apo-crystal structure of the polymerase from Thermococcus gorgonarius (Tgo) had been already solved [36] when this study was started.

The apo-structure of the variant TgoT exhibits several domain movements after comparison with the structure of WT Tgo DNAP [36] (PDB: 1tgo). Slight movements were observed for the N-terminal, the exonuclease, and the finger subdomains but much larger movements in the palm and the thumb subdomains were noted (Supplementary Figure S9). For instance, compared to their initial position in the Tgo DNAP structure, helices α19 and α20 of the thumb β subdomain of TgoT shift by almost 5.5 and 6 Å, respectively. Nevertheless, both structures are in the known and expected “open” conformation. While these movements could be due to differences in crystallogenesis conditions, they reveal the potential of flexibility in this multidomain enzyme. One could expect that a gain in flexibility in the TgoT variants would facilitate an efficient processing of non-natural substrates.

Currently, no structure of a DNAP from the B-family bound to a full-XNA primer or a full-XNA template has been solved. Indeed, only the A-family DNAP of Geobacillus stearothermophilus (Bst) was solved in the presence of both a fully modified FANA template strand and of a fully modified TNA template strand [33]. Both FANA and TNA heteroduplexes were shown to adopt B-form helical structures in this context. This observation came as a surprise for TNA, which strongly favors an A-helical geometry as observed for HNA [61,62]. Here, a model of the structure of TgoT_6G12 polB bound to an HNA-DNA duplex was obtained and shown to be stable by molecular dynamics (MD). Contrary to observations made with a TNA template in the presence of a polA polymerase, the HNA-DNA duplex is closer to an A-type helical geometry when bound to a polB polymerase.

The analysis of the model structure of TgoT_6G12 bound to an HNA-DNA duplex reveals several conformational changes. The polymerase variant stabilizes the HNA-DNA heteroduplex in ways that are different from what is observed for KOD polymerase, but the number of interactions made with the template strand remains roughly constant. On the other hand, the number of backbone interactions observed between side chain residues and the HNA primer strand is smaller than for TgoT_6G12 or KOD bound to a natural DNA primer strand. The main differences are observed after comparison of TgoT_6G12 bound to an HNA-DNA duplex and KOD bound to a DNA-DNA duplex. Most of mutated residues in TgoT_6G12 are residues that stabilize the template strand in its HNA-DNA complex but that interact with the primer strand in the KOD DNAP structure. Therefore, a consensus seems to emerge in favor of the accommodation of the template strand rather than an accommodation of the active site. At this stage, it looks like the primer HNA recognition is only indirect, namely it only involves the type of DNA double helix in which it is engaged.

Structural information available in both the ternary complex of TgoT_6G12 bound to a DNA-DNA duplex or bound to an HNA-DNA duplex provides an important framework for generating new HNA polymerase variants. For example, residues R606 or Y594 are good candidates for mutagenesis, as these residues contact the DNA primer phosphate backbone but not the HNA primer.

In addition to the in silico model, a new X-ray structure of a binary complex of TgoT_6G12 was solved, with a primer-template DNA elongated by two hCs. Strikingly, this structure reveals the presence of both an entry site for a backtracked form of the complex and a new dNTP binding site inside the polymerase, which we called the antechamber (Figure 8A). This new pocket is localized close to several positively charged amino acid groups of the finger subdomain (K487, R460, K464) and it is located close to and in front of the position −1 (entry site) that contains the second incorporated hC (Figure 8A). The triphosphate of an additional ddCTP molecule is visible in the electron density present in the antechamber, but the base and the sugar moieties of the ddCTP could not be modeled, presumably because of a high flexibility (Figure 8A). To confirm the existence of this antechamber, all our X-ray structures were re-analyzed in this region. The presence of this antechamber, which is reminiscent of a storage pocket, was actually observed in two other structures, namely the apo-structure of TgoT and the binary structure of TgoT_6G12, all grown in an excess of ddNTP.

This structure can also give insight into the mechanism of polymerization of an HNA polymerase. The observed backtracked complex of TgoT_6G12 elongated by 2 hC has translocated by one nucleotide backwards instead of forwards, and is similar to what has been suggested for multisubunit RNA polymerases [63].

It could be interpreted as an additional step (Figure 8B, step 4) in the catalytic cycle that would avoid having an empty site at position 0 at any time during the cycle. Additionally, it would allow the enzyme to “sense” if there is a possible incoming dNTP nearby. When the antechamber is fully occupied, the 3′ end would jump directly from position −1 to position +1 to leave the place for the incoming nucleotide (Figure 8B, step 5).

Alternatively, it could represent an obligatory step before sending the 3′ fraying end to the exonuclease catalytic site. The reason why this intermediate was trapped in the crystal state might be that it was obtained with an exonuclease-deficient mutant of Tgo polB, so that it would be artificially populated in a mutant that cannot drive the reaction irreversibly towards the removal of the 3′ nucleotide.

## 5. Conclusions

In summary, structural analysis of the crystal structure of an HNA-accepting DNAP variant bound to DNA-DNA duplex or a model of its interaction with an HNA-DNA heteroduplex provides insights into the importance of mutations for HNA substrate accommodation by the polymerase. The model of the ternary complex of TgoT_6G12 bound to an HNA-DNA heteroduplex initially modeled in the A-form was stable during a 500-ns-long MD simulation, and helps rationalize most of the mutated residues. Mutated residues are involved in the stabilization of the nascent hybrid HNA-DNA duplex by interacting essentially with the template strand of a heteroduplex in the A-helical geometry.

Finally, the structure of a post-chemistry complex after the incorporation to two hC nucleotides was solved and showed the existence of an antechamber for an additional dNTP as well as the possibility for the 3′ base to translocate by one nucleotide not only forward but also backward. Indeed, this structural model implies the existence of a 1 nt-backtracking mode already suggested in RNA polymerases [63] and seems to indicate that the kinetic model of this mutant is profoundly modified. Whether or not this is a general feature Tgo PolB mutants selected for HNA (or XNA) incorporation will require further studies.

## Figures and Tables

**Table 1:** Initial system composition for the Molecular Dynamics run, including hydrogen atoms.

Type	Number of Residues	Number of Atoms
Protein	755	12,435
DNA	13	414
HNA	11	378
Mn^2+^	2	2
Water	31,407	94,221
Na^+^	104	104
Cl^−^	85	85

**Table 2:** Diffraction data processing and refinement statistics.

	TgoT_Apo	TgoT_RT521_Apo	TgoT_6G12_Apo	TgoT_6G12_Binary	TgoT_6G12_Ternary	TgoT_6G12_Binary_hCTP
**Resolution range**	45.86–2.394 (2.48–2.394)	43.69–2.35 (2.434–2.35)	49.53–3.099 (3.21–3.099)	46.1–2.797 (2.897–2.797)	48.47–3.15 (3.263–3.15)	49.74–3.0 (3.107–3.0)
**Space group**	P 21 21 21	P 21 21 21	P 21 21 21	C 1 2 1	P 21 21 21	P 21 21 21
**Unit cell**	65.06 105.22 152.86 90 90 90	70.53 109.85 111.3 90 90 90	71.89 110.49 111.77 90 90 90	164.49 111.92 74.1 90 110.39 90	111.823 112.331 186.518 90 90 90	56.21 106.77 231.78 90 90 90
**Total reflections**	389,306 (33,918)	158,299 (14,944)	132,742 (13,197)	134,380 (13,082)	66,626 (3510)	320,482 (30,357)
**Unique reflections**	41,950 (3986)	36,486 (3526)	16,706 (1621)	31,062 (3015)	33,316 (1758)	28,837 (2829)
**Multiplicity**	9.3 (8.5)	4.3 (4.2)	7.9 (8.1)	4.3 (4.3)	2.0 (2.0)	11.1 (10.7)
**Completeness (%)**	99.28 (95.65)	99.08 (97.67)	99.37 (98.48)	99.57 (98.11)	80.49 (43.10)	99.88 (99.79)
**Mean I/sigma(I)**	11.45 (0.49)	7.69 (0.94)	8.72 (0.82)	7.18 (0.78)	17.89 (1.52)	9.21 (0.53)
**Wilson B-factor**	72.01	57.45	101.76	79.15	88.71	109.57
**R-merge**	0.1222 (4.034)	0.09902 (1.565)	0.193 (2.756)	0.1749 (1.737)	0.05693 (0.5029)	0.2038 (3.171)
**R-meas**	0.1294 (4.293)	0.1126 (1.793)	0.2067 (2.939)	0.1997 (1.981)	0.08052 (0.7112)	0.2136 (3.332)
**R-pim**	0.042 (1.442)	0.05237 (0.8555)	0.07307 (1.008)	0.09487 (0.9382)	0.05693 (0.5029)	0.06285 (1.006)
**CC1/2**	0.999 (0.287)	0.997 (0.486)	0.998 (0.552)	0.99 (0.319)	0.997 (0.53)	0.998 (0.292)
CC*	1 (0.667)	0.999 (0.809)	0.999 (0.843)	0.997 (0.696)	0.999 (0.832)	1 (0.672)
**Reflections Used in Refinement**	41,845 (3979)	36,401 (3516)	16,647 (1619)	31,027 (3011)	33,305 (1758)	28,816 (2825)
**Reflections used for** **R-free**	2096 (199)	1820 (177)	834 (81)	1550 (151)	1648 (93)	1442 (141)
**R-work**	0.2410 (0.4986)	0.225 (0.3986)	0.2616 (0.4530)	0.2308 (0.3985)	0.2319 (0.3545)	0.2253 (0.4123)
**R-free**	0.2618 (0.5556)	0.2469 (0.4188)	0.2649 (0.5542)	0.2422 (0.4173)	0.2472 (0.3294)	0.2429 (0.4564)
**CC(work)**	0.939 (0.387)	0.944 (0.675)	0.933 (0.535)	0.923 (0.517)	0.951 (0.567)	0.932 (0.418)
**CC(free)**	0.939 (0.173)	0.949 (0.713)	0.841 (0.435)	0.919 (0.566)	0.923 (0.435)	0.909 (0.530)
**Number of Non-hydrogen atoms**	6174	5759	6154	6429	12,971	6535
**Macromolecules**	6094	5641	6150	6336	12,907	6472
**Ligands**	23	118	4	13	64	63
**Protein Residues**	742	685	749	721	1475	737
**RMS(bonds)**	0.009	0.009	0.008	0.008	0.009	0.009
**RMS(angles)**	1.29	1.28	1.15	1.23	1.37	1.24
**Ramachandran Favored (%)**	97.54	96.75	97.05	98.04	96.80	97.26
**Ramachandran Allowed (%)**	2.46	2.95	2.82	1.96	3.20	2.74
**Ramachandran Outliers (%)**	0.00	0.30	0.13	0.00	0.00	0.00
**Rotamer Outliers (%)**	3.58	2.85	1.54	3.02	0.86	3.29
**Clashscore**	2.77	2.48	3.49	3.99	3.24	5.54
**Average B-Factor**	100.24	95.01	140.31	90.08	104.56	128.22
**Macromolecules**	100.33	95.73	140.33	90.37	104.66	127.63
**Ligands**	156.24	60.15	109.55	152.13	84.25	188.89

* is mathematically derived from CC_1/2_ using the relationship CC* = [2 CC_1/2_/(1 + CC_1/2_)]^1/2^ and provides an estimate of the CC that would be obtained between the final merged data set and the unknown true values that they are representing. Statistics for the highest-resolution shell are shown in parentheses.

## Supplementary Materials

The following are available online at https://www.mdpi.com/2218-273X/10/12/1647/s1, Supplementary Figure S1: HNA genetic polymer. Supplementary Figure S2: Sequence alignment of B-family DNA polymerases of known structures. Supplementary Figure S3: Pattern of interactions between TgoT_6G12 polymerase and the template/primer duplex, in the ternary complex. Supplementary Figure S4. Supplementary Figure S5: Pattern of interactions between TgoT_6G12 polymerase and the HNA-DNA heteroduplex. Supplementary Figure S6: TgoT_6G12 active site analysis upon binding to a HNA-DNA duplex. Supplementary Figure S7: Conformational changes of TgoT_6G12 that allow adaptation to a HNA-DNA duplex. Supplementary Figure S8: Comparison between the KOD and the TgoT_6G12 DNAP ternary structures bound to different types of duplexes. Supplementary Figure S9: Comparison between the structure of Tgo-wt DNAP and TgoT variant.
