# Supplementary material for: Structural Studies of HNA Substrate Specificity in Mutants of an Archaeal DNA Polymerase Obtained by Directed Evolution
Source: Biomolecules. 2020 Dec 8;10(12):1647. doi: 10.3390/biom10121647 (PMC7763228; doi:10.3390/biom10121647)
Supplement: Supplementary file 1 [file biomolecules-10-01647-s001.pdf]

Supplementary Figures

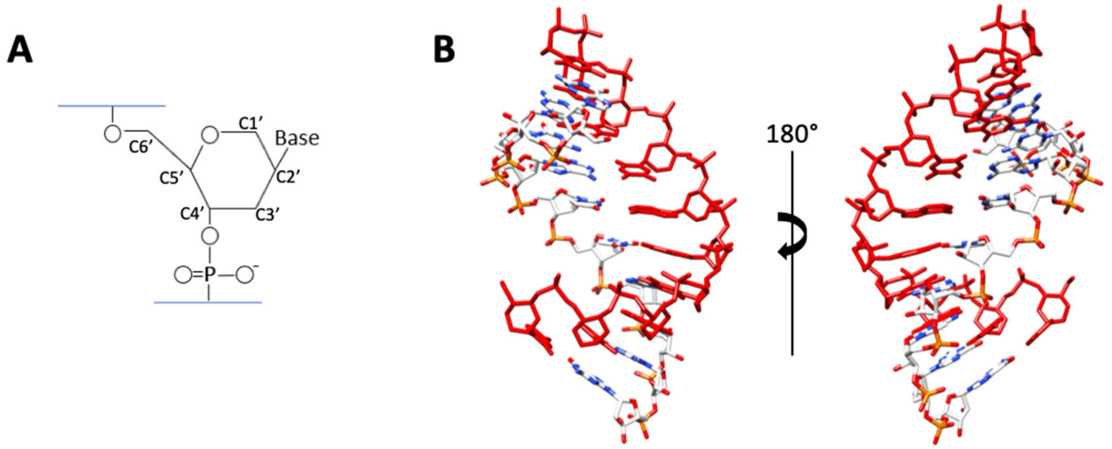

Supplementary Figure S1: HNA genetic polymer. (A) Chemical structure of 1,5-anhydrohexitol nucleic acid (HNA) repeating unit. (B) Crystal structure of a HNA-RNA hybrid duplex (PDB: 2BJ6). The HNA strand is labeled in red. The RNA strand is colored by atom type.

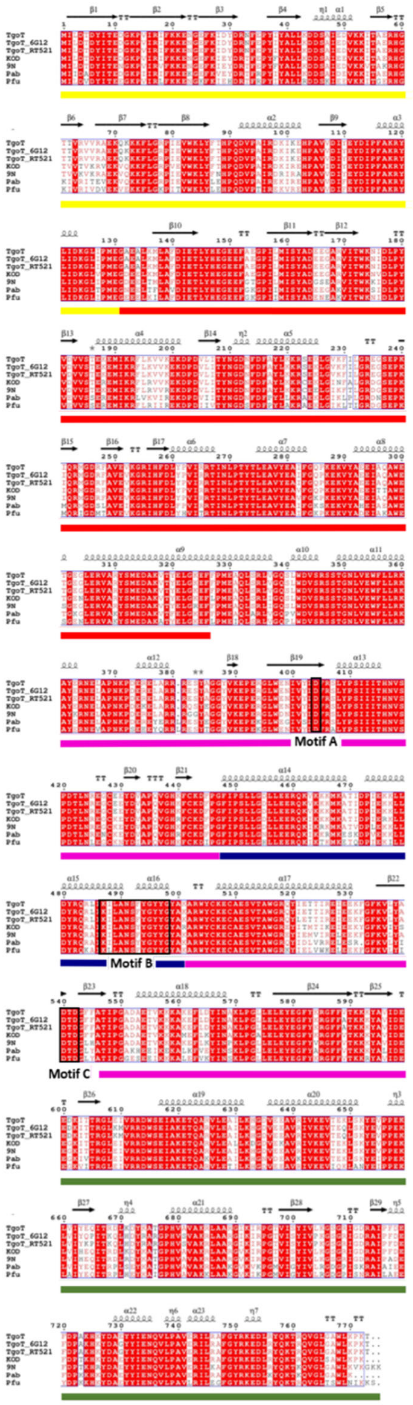

Supplementary Figure S2: **Sequence alignment of B-family DNA polymerases of known structures.** The alignment was made using Blast, Multalin and ESPript [64]. The secondary structure of TgoT is indicated on top of the alignment with helices (squiggles), β–strands (arrows) and turns (TT letters). Each domain is labeled, under the sequence alignment, with colored rectangles. The domains are labeled as follows: N-terminal domain (yellow), 3'-5' exonuclease domain (red), palm domain (magenta), fingers domain (blue) and thumb domain (green). The Motifs A, B and C are boxed in black. Abbreviations: TgoT, TgoT_6G12 and TgoT_RT521, *Thermococcus gorgonarius* variants; KOD, *Thermococcus kodakarensis* polymerase; 9N, *Thermococcus sp. 9°N-7* polymerase; Pab, *Pyrococcus abyssi* polymerase; Pfu, *Pyroccocus furiosus* polymerase.

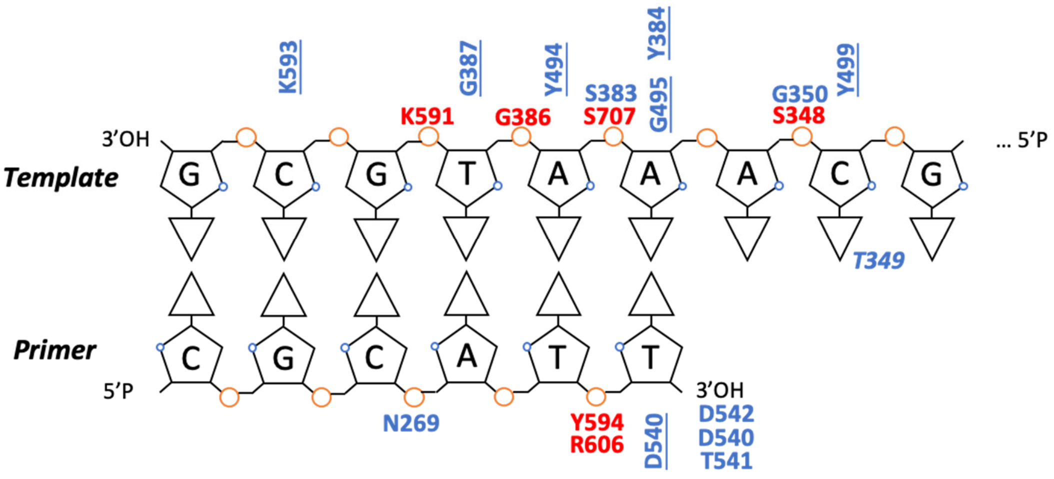

Supplementary Figure S3: Pattern of interactions between TgoT_6G12 polymerase and the template/primer duplex, in the ternary complex. Residues are labeled and colored according to the strength of the interaction, as defined in Figure 4. Interactions between the enzyme and the phosphate backbone of the DNA are labeled above, for the template strand, and below, for the primer strand, each phosphate (represented by an orange circle). Interactions between the enzyme and the ribose moieties are underlined and vertical.

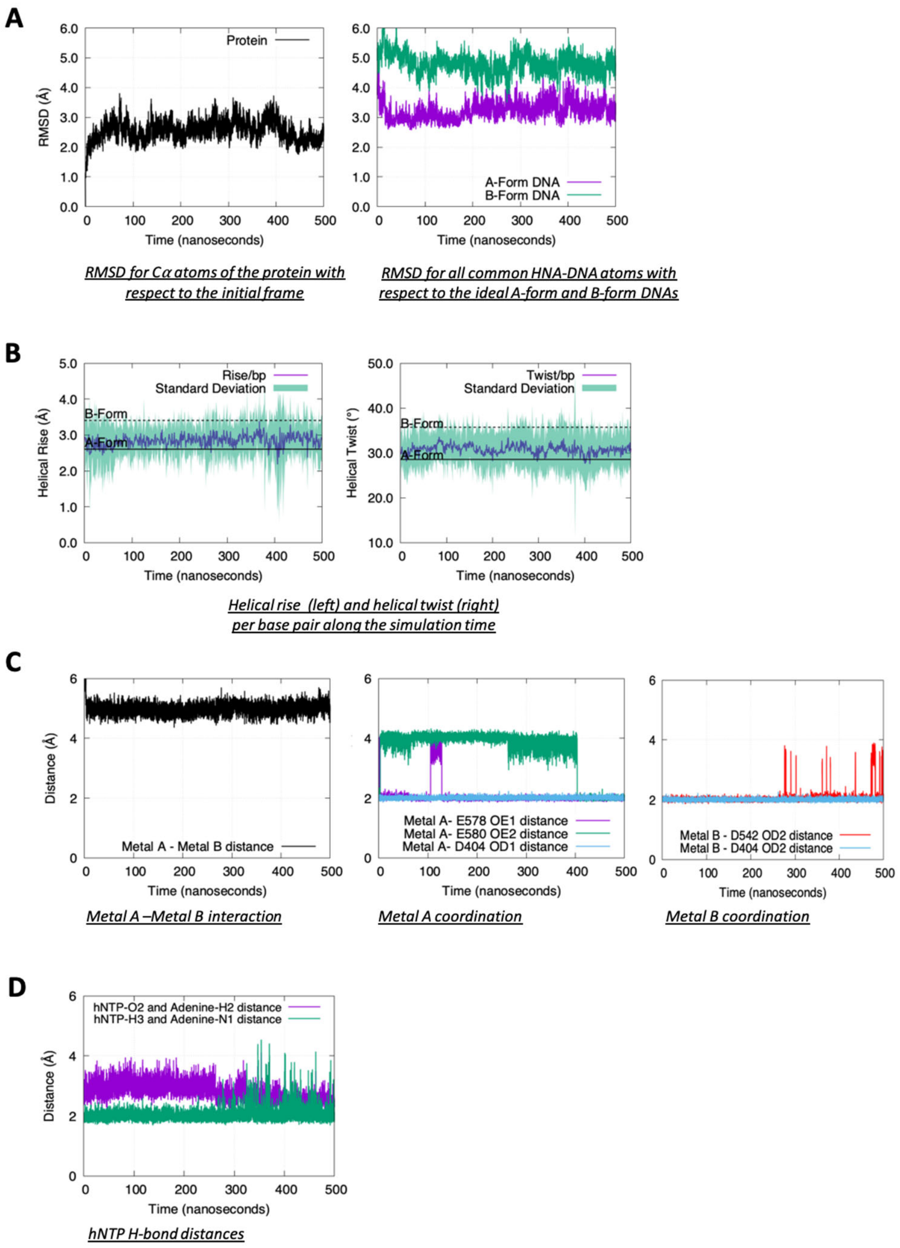

Supplementary Figure S4: MD simulation of the HNA-DNA duplex bound to TgoT_6G12. (A) Left: A plot of the global Cα RMSD as a function of time. Right: RMSD for all common HNA-DNA atoms using A-form and B-form DNAs as reference structures. (B) Helical rise (left) and helical twist (right) per base pair along the simulation time. Horizontal black dashed lines indicate values for the ideal B-form DNA, while solid black lines highlight the ideal A-form DNA values. (C) Left: distances of the first Mn^{2+} (Metal A) and the second one (Metal B) across the trajectory. Middle: distances of Mn^{2+} (Metal A) to residues E578, E580 and D404, labeled in purple, green and light blue, respectively. Right: distances of Mn^{2+} (Metal B) to residues D542 and D404, labeled in red and light blue, respectively. (D) Left: H-bond distances of the bound hNTP to the Adenine nucleotide of the DNA in acceptor-donor atoms, with hNTP-O2 and Adenine-H2 distance in purple, hNTP-H3 and Adenine-N1 distance in green.

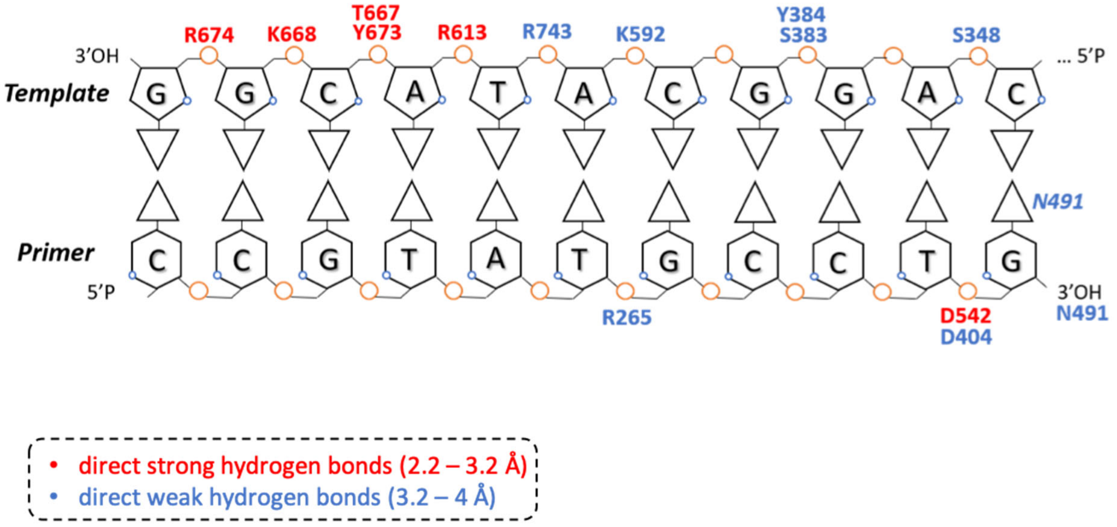

Supplementary Figure S5: Pattern of interactions between TgoT_6G12 polymerase and the HNA-DNA heteroduplex. Residues are labeled and colored according to the strength of the interaction as defined in Figure 4. Interactions between the enzyme and the phosphate backbone of the DNA are labeled above, for the template strand, and below, for the primer strand, each phosphate (represented by an orange circle). Interactions between the enzyme and the ribose groups are underlined and vertical. Interactions between the polymerase and the nucleotide base are in italics.

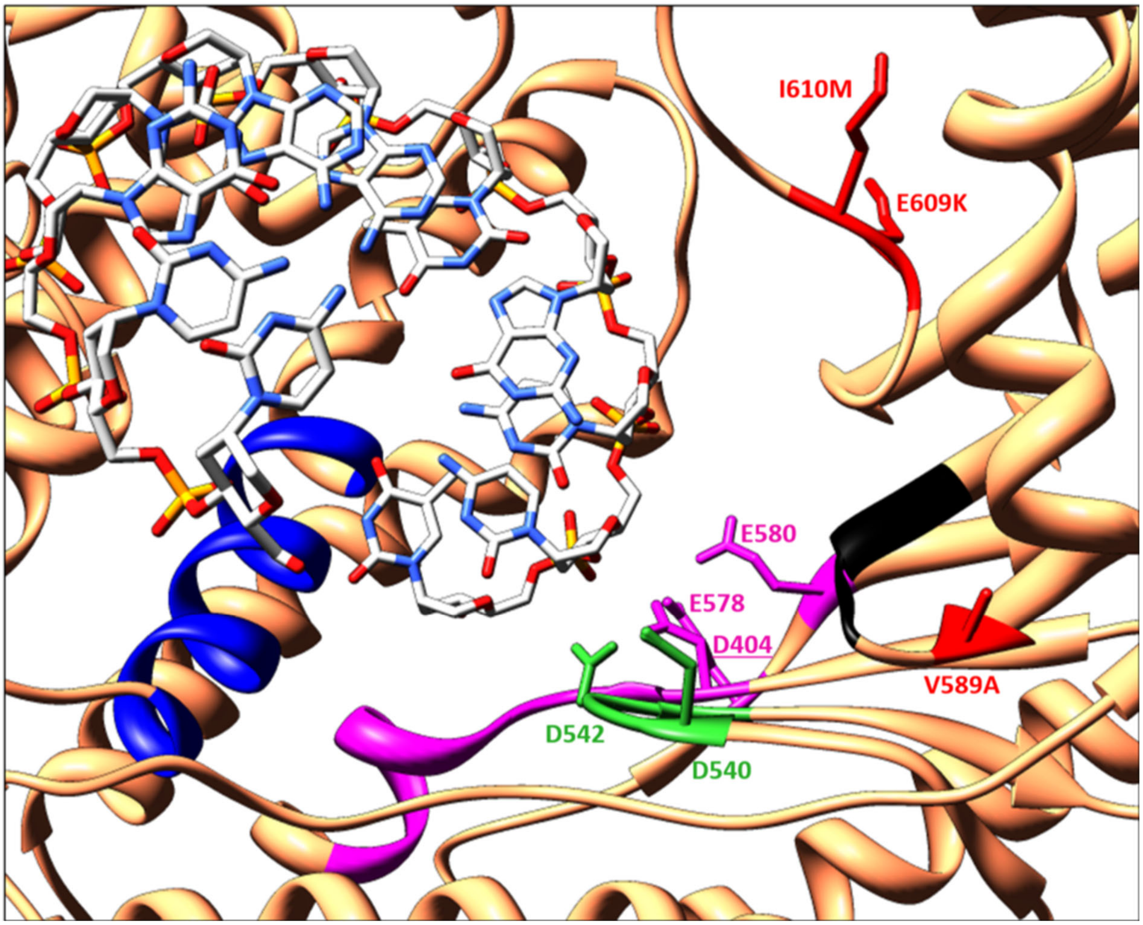

Supplementary Figure S6: TgoT_6G12 active site analysis upon binding to a HNA-DNA duplex. Model of TgoT_6G12 active site with a bound HNA primer strand. Mutated residues that allow HNA binding are colored in red. Representative motifs are labeled. The $Kx_3NSxYGx_2G$ B-motif in the finger domain, is colored in dark blue. The A-motif is colored in magenta and the catalytic aspartate residue is underlined. The C-motif (DTDG) of the palm domain, is colored in green and the KKKY motif of the thumb domain is colored in dark. The 3’OH of the primer makes contacts with residues of the palm domain (D404, D540, D542, E578 and E580), labeled in magenta.

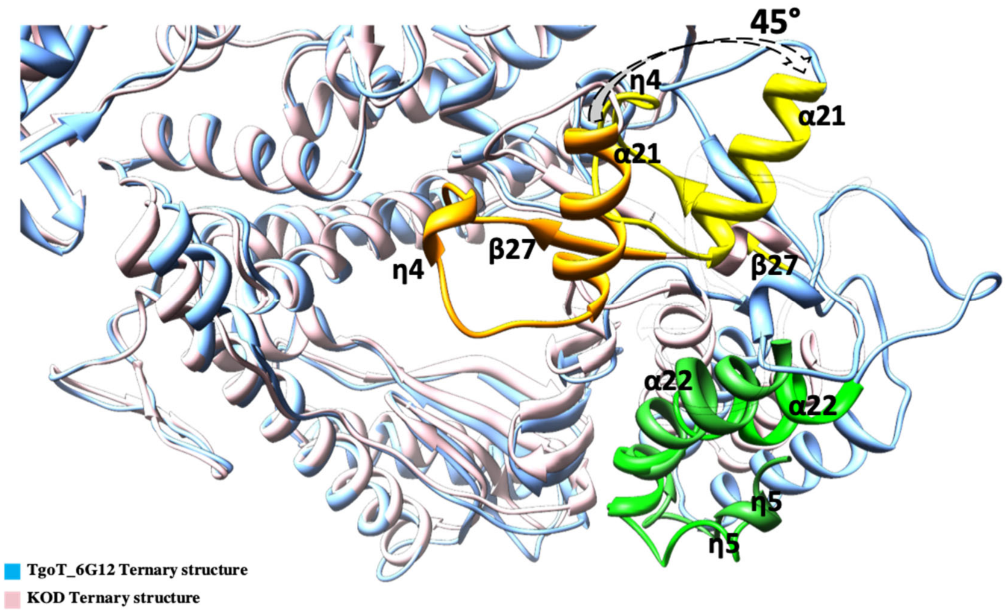

Supplementary Figure S7: Conformational changes of TgoT_6G12 that allow adaptation to a HNA-DNA duplex. Superimposition of the thumb domains of KOD in a ternary complex and of TgoT_6G12, also in a ternary complex. Missing parts of the thumb domain of TgoT_6G12 were modeled from 5VU9 structure. The X-ray ternary structure of TgoT_6G12 is colored in light blue. The X-ray ternary structure of KOD is colored in light pink. The region from amino acids 664 to 688 is labeled in yellow for TgT_6G12 and in orange for KOD. Helix η5 and α–helix 22 are labeled in light and dark green, respectively, in TgoT_6G12 and in KOD ternary complexes.

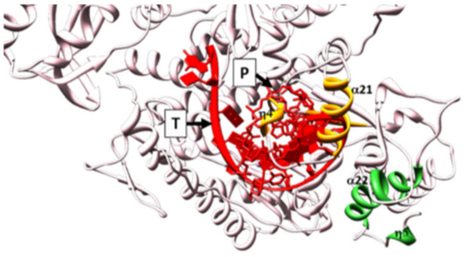

KOD ternary complex bound to a HNA-DNA duplex

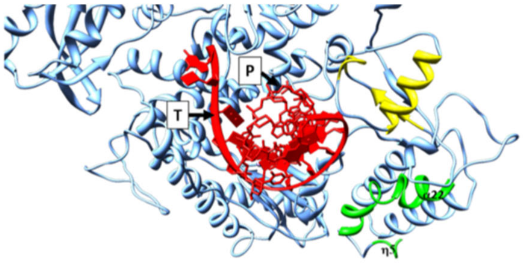

TgoT_6G12 ternary complex bound to a HNA-DNA duplex

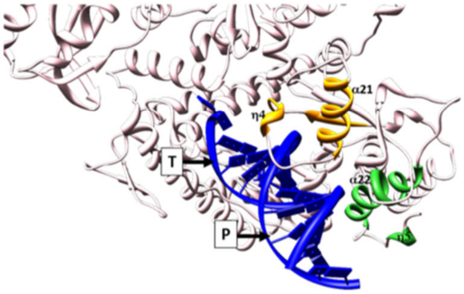

KOD ternary complex bound to a DNA-DNA duplex

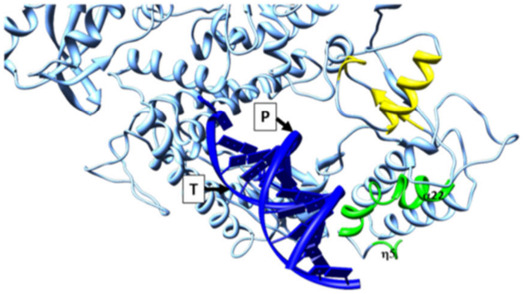

TgoT_6G12 ternary complex bound to a DNA-DNA duplex

Supplementary Figure S8: Comparison between the KOD and the TgoT_6G12 DNAP ternary structures bound to different types of duplexes. (upper panel) The HNA-DNA heteroduplex, as modeled for the *in silico* structure of TgoT_6G12 was superimposed with the X-ray structure of KOD and TgoT_6G12 ternary complexes. The primer and template strands are indicated. The position of the HNA-DNA would induce a steric clash with the thumb domain of KOD (on the left). By contrast, the thumb domain of TgoT_6G12 is better suited to the position of the heteroduplex (on the right). (B) (Down panel) While the region from amino acids 664 to 688 (in orange) interacts with the primer strand in KOD ternary complex (on the left), this same region (in yellow) shifts away from the DNA-DNA duplex in TgoT_6G12 (on the right). Helix η5 and α–helix 22 are colored in light and dark green, respectively, in TgoT_6G12 and in KOD ternary complexes.

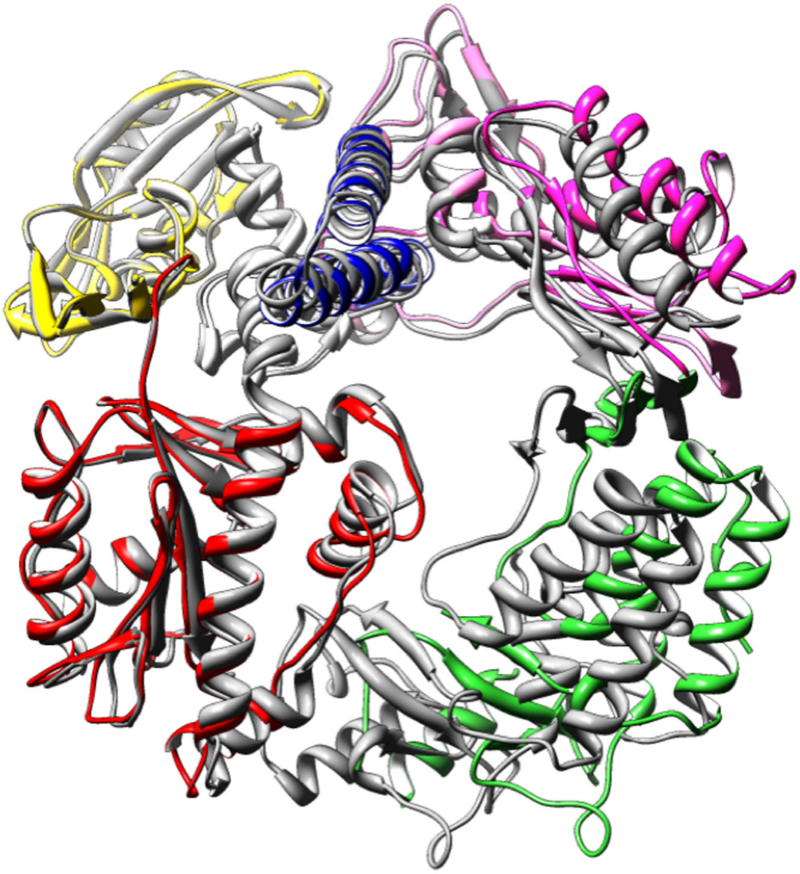

Supplementary Figure S9: Comparison between the structure of Tgo-wt DNAP and TgoT variant. Overview and superimposition of the X-ray structures of Tgo DNAP (PDB: 1tgo) and of TgoT variant. Tgo DNAP is colored in gray. The domains of TgoT variant are colored as follows: N-terminal domain (yellow), 3′-5′ exonuclease domain (red), palm domain (light and dark magenta for the N-terminal and the C-terminal parts, respectively), fingers domain (blue) and thumb domain (green). An interhelical segment between the exonuclease and the palm domain is labeled in light grey. Large movements between palm and thumb domains of both structures are observed.
